# Intratumor microbiome features reveal antitumor potentials of intrahepatic cholangiocarcinoma

**DOI:** 10.1080/19490976.2022.2156255

**Published:** 2022-12-23

**Authors:** Xiaoqiang Chai, Jie Wang, Huanping Li, Chao Gao, Shuangqi Li, Chuanyuan Wei, Jianhang Huang, Yingming Tian, Jian Yuan, Jiacheng Lu, Dongmei Gao, Yimin Zheng, Cheng Huang, Jian Zhou, Guoming Shi, Aiwu Ke, Feng Liu, Jia Fan, Jiabin Cai

**Affiliations:** Institutes of Biomedical Sciences, Department of Liver Surgery and Transplantation, Liver Cancer Institute, Zhongshan Hospital, Key Laboratory of Carcinogenesis and Cancer Invasion of Ministry of Education, Key Laboratory of Medical Epigenetics and Metabolism, Fudan University, Shanghai, China

**Keywords:** ICC, bacteria, *P. fungorum*, metabolomics, amino acid metabolism

## Abstract

Intrahepatic cholangiocarcinoma (ICC) is a rare malignancy with a high prevalence in China. This study aimed to characterize the ICC tissues’ bacterial metagenomics signature and explore its antitumor potential for cancer. In this study, 16S rRNA sequencing was carried out on 99 tissues to characterize the features of intratumoral microbiota, followed by single-cell RNA sequencing (scRNA-seq) and multilevel validation. The presence of microbial DNA in tissues was determined using staining, fluorescence in situ hybridization (FISH), and transmission electron microscopy (TEM). A Gram-positive aerobic bacterium, identified as *Staphylococcus capitis*, was cultured from fresh tissues. Meanwhile, scRNA-seq showed that intratumoral bacteria could be present in multiple cell types. Using 16S rRNA sequencing, we identified a total of 2,320,287 high-quality reads corresponding to 4,594 OTU (operational taxonomic units) sequences. The most abundant bacterial orders include *Burkholderiales, Pseudomonadales, Xanthomonadales, Bacillales* and *Clostridiales*. Alpha and Beta diversity analysis revealed specific features in different tissues. In addition, the content of *Paraburkholderia fungorum* was significantly higher in the paracancerous tissues and negatively correlated with CA199 (Carbohydrate antigen199) levels. The results of in vitro and in vivo experiments suggest that *P. fungorum* possesses an antitumor activity against tumors. Metabolomics and transcriptomics showed that *P. fungorum* could inhibit tumor growth through alanine, aspartate and glutamate metabolism. We determined the characteristic profile of the intratumoral microbiota and the antitumor effect of *P. fungorum* in ICC.

## Introduction

Cholangiocarcinoma (CHOL) is a kind of malignant tumor occurring in the biliary system of the liver due to carcinoma of the biliary epithelium, which has less incidence than hepatocellular carcinoma (HCC)^1^. As a common type of CHOL, intrahepatic cholangiocarcinoma (ICC) has a high incidence in China and Southeast Asia, with the disease having a degree of high malignancy and poor prognosis.

Recent studies have found that human commensal microbes are closely associated with complex diseases such as mental disorders,^2^ cardiovascular diseases,^3^ and tumors.^4^ Studies have shown the significance of that intestinal microbiota in maintaining the intestinal mucosal barrier and promoting the development and maturation of the immune system.^5,6^ An association exists between melanoma patients with different gut microbial compositions and different treatment outcomes.^7^ Microbial communities have been shown to parasitize the human liver and bile ducts, and they can affect liver metabolism, immune environment, and disease status.^4,8^ Clinical studies have found that intestinal microbiota imbalance is associated with the progression of nonalcoholic steatohepatitis (NASH) and cirrhosis.^9–11^ Another study reported that the intestinal microbiota and a functional Toll-like receptor 4 (TLR4), are required for hepatic fibrogenesis.^12^ Both clinical and animal studies have shown that microbial communities can stimulate hepatic inflammatory responses and liver fibrosis and induce IL-6 production, which in turn promotes the development of liver tumors.^13^ However, relatively few studies have been conducted on the relationship between microorganisms and bile duct cancer. Epidemiological studies have shown that *Helicobacter pylori* in bile is a risk factor for increased risk of bile duct cancer.^14^ *H. pylori* located in the biliary tract can activate the NFKB signaling pathway, increase the transcriptional expression of vascular endothelial growth factor, and promote tumor angiogenesis.^15^ Therefore, a good understanding of how intrahepatic microbiota influences the development, progression and treatment of liver and bile duct disease deserves further investigation.

With the development of metagenomic technology, researchers have discovered many novel microbial taxa in different environments. Metagenomic sequencing technology has a wide range of promising applications in clinical diagnosis, such as metagenomic sequencing of stool or blood samples to diagnose infectious diseases and malignant tumors.^16^ A team of Israeli scientists found that in tumors such as brain, bone, and breast cancers, bacteria are present in most tumors and their adjacent normal tissues, and different types of tumors even have their unique community structure. However, whether and how bacteria within tumor cells contribute to tumorigenesis, progression, and response to treatment requires further in-depth study.^17^ Several researchers have studied the microbiome of tissues from patients with bile duct cancer associated with *Schistosoma haematobium*, but they have not further explored the biological functions of bacteria in the tissues.^18^

In this study, we sequenced 16S rRNA from the tissues of ICC patients and quantified the bacteria in the tissues of ICC patients using computational biology methods. Meanwhile, the presence of intracellular bacteria in bile duct cancer tissues was verified by bacterial culture and transmission electron microscopy (TEM) experiments. Moreover, we verified the biological function of *Paraburkholderia fungorum* via in vitro and in vivo experiments, and omics data demonstrated that it may affect tumor growth by participating in amino acid metabolic pathways. All in all, our study provides a comprehensive overview of the microbiome with confirmation of the anti-tumor effects of bacteria and suggests potential approaches for ICC therapy.

## Results

We collected 94 clinical samples (42 paired tumor (T) and paracancerous (P) tissues, plus unpaired 3 tumor tissues and 7 paracancerous tissues) from 52 ICC patients. The clinical and biochemical features of ICC patients are presented in Table 1.

**Table 1.** Clinical information of ICC patients enrolled in 16S sequencing.

clinical factor	Sample Information Statistics
Patients	ICC (n = 52)
T/N/M Staging	II (n = 32), III (n = 18), III (n = 2)
Alpha-fetoprotein (AFP)	5.9 ± 12.6
Albumin (albumin)	43.1 ± 3.5
Total bilirubin (TBil)	13.5 ± 7.7
Alanine aminotransferase (ALT)	48.0 ± 91.0
Gamma-glutamyl transferase (GGT)	140.9 ± 221.2
Glycoantigen 199 (CA19-9)	953.6 ± 1841.7
White blood cells (WBC)	6.9 ± 2.8
Hepatitis B virus surface antigen (HBsAg)	+ (n = 12), – (n = 40)
Hepatitis B virus surface antibody (HBsAb)	<2.0 (n = 21), >2.0 (n = 31)
Hepatitis B virus e antigen (HBeAg)	+ (n = 0), – (n = 52)
Hepatitis B virus e antibody (HBeAb)	+ (n = 28), – (n = 24)
Hepatitis B virus core antibody (HBcAb)	+ (n = 41), – (n = 11)
Hepatitis B virus (HBV-DNA)	+(n = 6), – (n = 46)
Hepatitis C virus antibodies (HCVAb)	+(n = 0), – (n = 52)

Numerical information is expressed as average ± stdev; +, positive; -, negative; n in parentheses is the number of cases.

### 16S rRNA sequencing reveals distinct microbiome features in ICC patients

**Figure 1.**
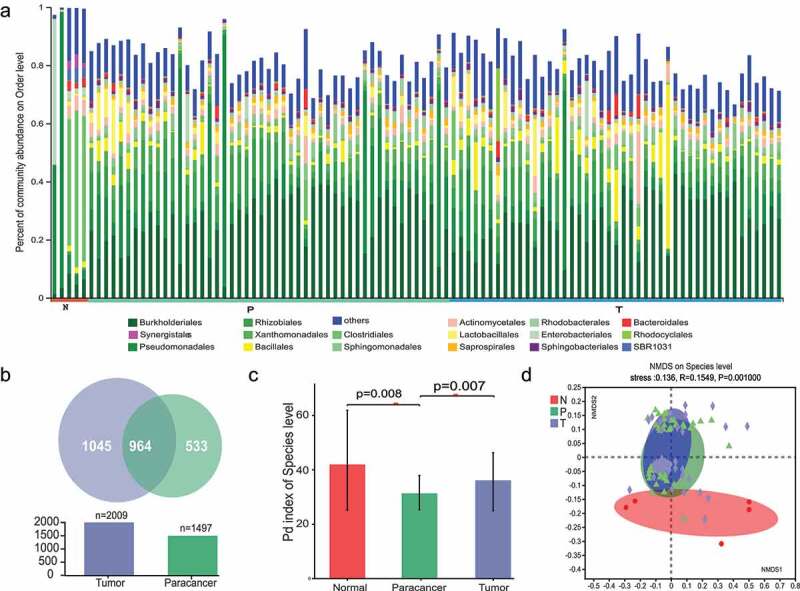
Analysis of the composition of bacterial microbiota in ICC tissues. (a) Composition features of the microbiota in each group at the order level. The color was set by phyla using different shades of color for the different orders within a phylum. (b) Relationship between tumor and paracancerous microbiota at the species level. (c) Comparison of differences in the alpha diversity index (PD index) of microbiota in groups N, P and T. (d) Beta diversity index reflecting the composition of the microbiota between groups N, P and T. PD: Phylogenetic diversity; N: normal liver tissues; P: ICC paracancerous tissues; T: ICC tumor tissues.

To remove the interference of contaminating bacteria introduced during the experimental manipulation, negative controls were used in the DNA extraction and PCR amplification process. By analyzing the 16S rRNA sequencing data, we found that the negative control samples contained a variety of bacteria (Supplementary Table S1; Supplementary Fig. S1). We used Decontam^19^ to remove contaminating bacteria in the operating environment and to identify the specific microbial composition of ICC patient tissues. After removing contaminating bacteria from the negative control and filtering out individual low-quality samples, a final total of 2,320,287 sequencing reads with an average length of 413 bp was obtained. These reads correspond to 4,594 OTU (operational taxonomic units) sequences with species taxonomic levels corresponding to 266 species, 602 genera, 282 families, 160 orders, 98 classes, 33 phyla, and 1 kingdom (Supplementary Table S2). Among them, bacteria with high abundance included *Burkholderiales, Pseudomonadales, Xanthomonadales, Bacillales, Clostridiales*, and *Sphingomonadales* (Figure 1a). The results of this study show an overall similar pattern of microbial composition between carcinoma and adjacent tissues. The differences in microbial composition between tumor and paracancerous were greater relative to normal liver tissues.

Given the significant differences in the tumor microenvironment between normal liver tissues and paracancerous and cancerous tissues, we explored the compositional diversity of bacterial microbiota in these three groups of samples. A Venn diagram of the composition of the microbiota in the tissues showed that ICC cancer tissues had a greater variety of microorganisms (Figure 1b). At the OTU level, the number of bacterial consortiums shared by the T and P groups was 964. The calculation of the spectral diversity index (PD: Phylogenetic diversity) showed that the Alpha diversity of bacterial microbiota was significantly higher in ICC tumor tissues than in paracancerous tissues (Figure 1c). The selected normal tissue was derived from patients with liver metastases from intestinal cancer or hepatic hemangioma, which we speculated to be more different from the pathological background of ICC cancer and paracancerous tissue, and therefore did not include the normal group of differential bacteria in the subsequent comparison step. In summary, the PD index results for the three groups of samples indicated that there were differences in the composition of the microbiota between the groups.

We next calculated the Beta diversity of community species using the NMDS analysis (non-metric multidimensional scaling) method, which characterizes the similarities or differences in community composition among different subgroups. By calculation, we observed that there was a significant difference in beta diversity among the three groups of samples (groups N, P and T). However, there was also a great similarity between group P and group T relative to group N (Figure 1d). As can be seen from the figure, there is more overlap between tumor and paracancerous groups than the normal group, which is because clinical samples from cancer and paracancerous are paired, i.e., tumor and paracancerous tissues are usually selected during clinical sampling to facilitate subsequent comparisons.

### FISH, bacterial culture, and TEM experiments reveal the presence of live and intracellular bacteria in tissues

**Figure 2.**
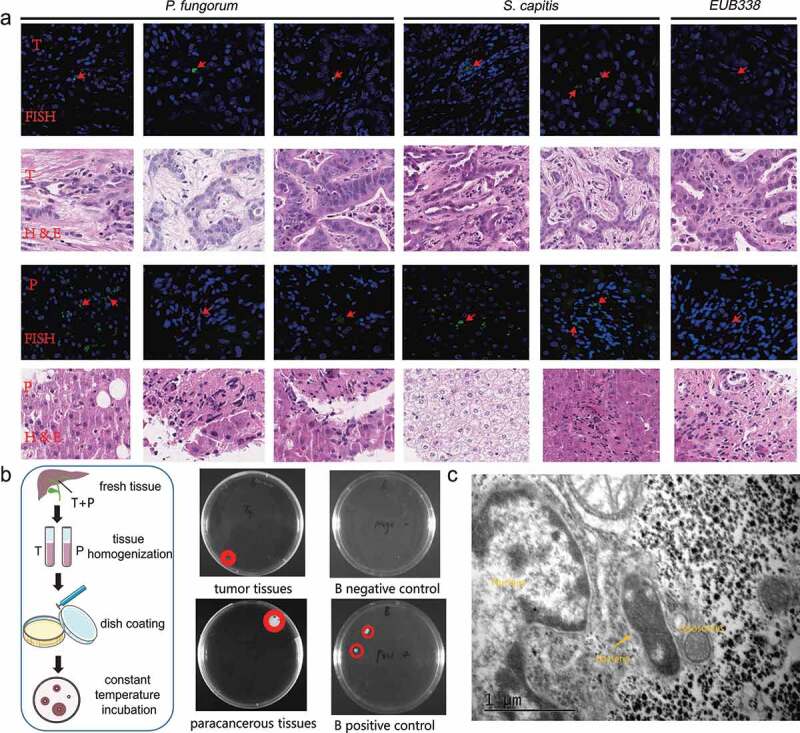
Validation experiments show the presence of bacteria in the tissues. (a) Results of FISH fluorescence staining and HE section staining of ICC tissues. The green signal indicates the synthetic FISH probe and the red signal indicates the positive probe signal (EUB338). The red arrow indicates the spot of the signal in the slices. T: tumor tissue slice; P: paracancerous tissue slice. (b) Fresh ICC patient tissue (tissue obtained shortly after surgery) was used to homogenize and culture live bacteria. The flowchart on the left shows the basic process of culturing bacteria from fresh tumor tissues. The diagram on the right shows the microbial colonies cultured from fresh tissues. Pure culture medium was used as a negative control, and wipes from the sink or door handle environment were used as a positive control. ‘B’ denotes that we used Brain heart infusion (BHI), which is a growth medium for growing microorganisms. (c) TEM experiments show the existence of bacteria in ICC tumor tissues (the locations marked with yellow arrows denote bacteria).

Previously, by 16S rRNA sequencing, the presence of bacterial DNA in ICC tissues was initially determined. Then, DNA oligonucleotide probes were designed and FISH was used to explore the presence of target bacterial DNA in the tissues. In our study, DNA probes for *Klebsiella pneumoniae, Pseudomonas azotoformans, S. capitis* and *P. fungorum* were designed and hybridized to tissue sections of ICC. H&E staining and FISH fluorescence staining revealed the presence of DNA in the ICC tissues (Figure 2a; Supplementary Fig. S2A).

Given the presence of bacterial DNA in tumor tissues and combined with reports from other researchers on microbes in tissues, we hypothesize that live bacteria are present in fresh tumor tissues and can be isolated by in vitro culture. In this study, fresh tumor tissues were taken for homogenization, and then the tissue homogenate was coated onto plates and placed in a thermostat for incubation. By incubation, it was observed that colonies were present in the plates, indicating the presence of viable bacteria in the tumor and paracancerous tissues (Figure 2b). Multiple colonies were picked to separately identify them by mass spectrometry, and the presence of *Staphylococcus capitis* in fresh tumor tissue of ICC was found, with the genus being present in our 16S rRNA sequencing data.

Using the bacterial culture experiments described above, we determined the presence of live bacteria in fresh tumor tissues, but could not visualize the bacterial morphology. As a result, it was discovered using TEM experiments that intracellular bacteria were present in tumor tissue and paracancerous tissue and that they could be encapsulated in lysosomes (Figure 2c; Supplementary Fig. S2B). Given that lysosomes can break down substances entering the cell from the outside, we presume that the bacteria entering the cell will be lysed by the lysosome, while the part that is not completely lysed can be observed by electron microscopic imaging. The figure shows that the bacteria have been digested by the lysosomes, but their outlines are still visible. From the distribution position of the bacteria, we observed that some of the bacteria are located outside the cell, but others are located inside the cell, which is consistent with the findings of others.^17^

### Single-cell transcriptome data shows the enrichment of bacterial DNA transcription products in immune cells

**Figure 3.**
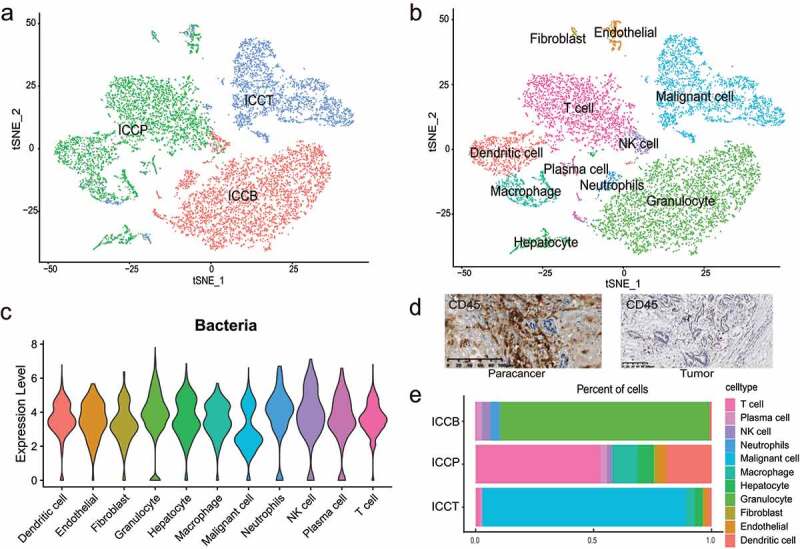
scRNA-seq analysis reveals the presence of bacterial RNA in each cell type. (a) t-SNE plot showing cell origins by color. (b) t-SNE plot showing cell clusters including fibroblasts, endothelial cells, malignant cells, hepatocytes, granulocytes, neutrophils, dendritic cells, macrophages, NK cells, plasma cells and T cells. (c) Violin plot showing the levels of bacterial RNA content in each cell type. The RNA content is reflected by sequencing reads count. (d) CD45 immunohistochemical staining shows an increased abundance of immune cells in the paracancerous tissue. (e) The proportion of cell counts of each cell type in each sample. B, plasma; P, paracancerous tissue; T, tumor tissue.

In combination with reports by others, the presence of intracellular bacteria was verified utilizing bacterial culture and TEM experiments. However, it was not able to fully depict the changes in the distribution of bacteria in various cell types. As such, we analyzed ICC scRNA-seq data from three tissue samples (one each for tumor, paracancerous, and plasma tissues) from ICC patients, yielding a total of 11,357 cells for subsequent cluster analysis (Figure 3a). The tSNE clustering method^20^ was used to classify all cells into 11 cell clusters, and these 11 cell classes were annotated as fibroblast, endothelial, hepatocytes, malignant cells, and multiple immune cells by reviewing marker genes^21–25^ in the literature (Figure 3b; Supplementary Fig. S3). Unlike the conventional human scRNA-seq data analysis, we added the number of bacterial reads to the initial matrix, and the results showed that bacterial RNA was present not only in parenchymal cells such as bile duct cells and hepatocytes but also in a variety of immune cells like macrophages and NK cells (Figure 3c).

Next, the bacterial transcription products in each cell of the sample were calculated using scRNA-seq analysis. The results showed that bacteria in plasma were enriched in granulocytes, which belong to white blood cells. In paracancerous tissues, bacterial transcription products were most abundant in T cells, perhaps because T lymphocytes would be actively involved in the immune response in paracancerous tissues (Figure 3d). The most predominant cell type in the tumor tissue is the malignant cell, which mainly includes cancerous hepatocytes and bile duct cells, and therefore most bacteria in tumor are enriched in this cell type (Figure 3e). Differential expression results showed that bacteria were more abundant in malignant cells of cancerous tissue than in paracancerous tissue, while bacteria were less abundant in cancerous tissue T cells than in paracancerous tissues. It is considered that immune cells, especially T cells, can act directly on malignant tumor cells, and both types of cells have higher levels of bacteria. Moreover, a recent study on melanoma found that bacterial protein peptide fragments could bind to tumor cells and thus be recognized by T cells.^26^ Therefore, we speculate that they can be directly involved in the body’s immune response.

### *Clinical factor analysis shows a negative correlation between* Burkholderia *and CA199 levels*

**Figure 4.**
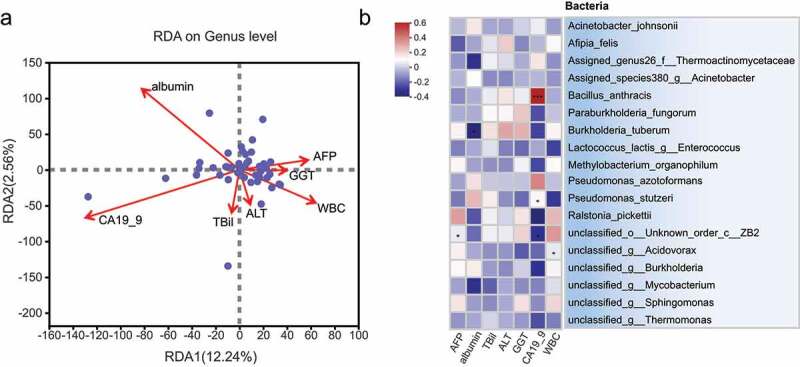
Environmental factor analysis showing the correlation of bacterial abundance with clinical data. (a) RDA/CAA environmental factor analysis shows the degree of influence of clinical factors on microbiota composition, with individual points in the figure indicating tumor tissues and arrows indicating clinical factors. (b) Correlation analysis of clinical factors with the abundance of microbiota in tissues. The horizontal axis indicates the common clinical factors in ICC, including alpha-fetoprotein (AFP), albumin (albumin), total bilirubin (TBil), alanine aminotransferase (ALT), gamma-glutamyl transferase (GGT), glycoantigen 199 (CA19-9) and White blood cells (WBC). The vertical axis indicates the bacteria identified by 16S rRNA. Different colors in the graph indicate the magnitude of the correlation coefficients; * 0.01 < P ≤ .05, ** 0.001 < P ≤ .01, *** P ≤ .001.

Then, clinical information of patients was collected to explore the relationship between intratumoral microbial abundance and clinical factors. Considering the correlation between clinical factors, a preliminary screening of clinical information was performed before performing a clinical factor association analysis. The clinical information selected for the study comprised AFP, albumin, total bilirubin, ALT, GGT, CA199 and WBC. The RDA/CAA environmental factor analysis showed that the cancer antigen CA199 had the greatest effect on tumor tissue bacteria, followed by albumin and leukocytes (Figure 4a). Two of these clinical factors, CA199 and albumin, were positively correlated, while CA199 was negatively correlated with leukocytes and methemoglobin. By calculating the correlation between intratumoral microbial classification and clinical factors, the amount of *Bacillus anthracis* and *P. azotoformans* was found to be positively correlated with CA199 (Figure 4b). Comparatively, the amounts of *Burkholderia tuberum* and *P. fungorum* were negatively correlated with CA199 and were associated with high expression of this bacterium in ICC paracancerous tissues, from which we speculated that they may have antitumor properties.

### Functional predictions indicate that bacterial microbiota may be associated with amino acid biosynthesis

**Figure 5.**
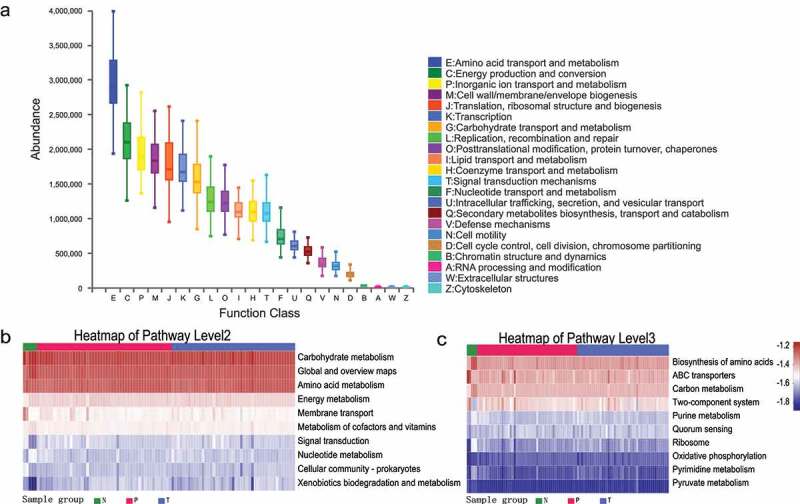
Functional prediction analysis of bacterial microbiota. (a) PICRUSt2 combined with the egNOG database to predict the function of bacterial microbiota in tissues. PICRUSt2 combined with the KEGG database to predict the function of bacterial microbiota in tissues, showing the results of the KEGG pathway in Level 2 (b) and Level 3 (c).

Based on the 16S amplicon sequencing data combined with the eggNOG and KEGG databases, we predicted the biological functions of the bacteria (Figure 5). In combination with the eggNOG database, the functions of bacteria were predicted to be mainly involved in metabolism, with amino acid metabolism accounting for the highest abundance (Figure 5a; Supplementary Table S3). Similarly, the 16S rRNA sequencing data combined with KEGG functional predictions indicated that bacterial colony function is primarily related to the pathway of amino acid and nucleotide metabolism of carbohydrates (Figure 5b; Supplementary Table S3). Combined with information from KEGG data at a deeper level (3rd Level), we could further confirm the association of bacteria with the biosynthesis of amino acids, purine metabolism, pyrimidine metabolism, and pyruvate metabolism (Figure 5c; Supplementary Table S3).

### *Differential expression analysis reveals higher biomass of* Burkholderia *in paracancerous tissues*

**Figure 6.**
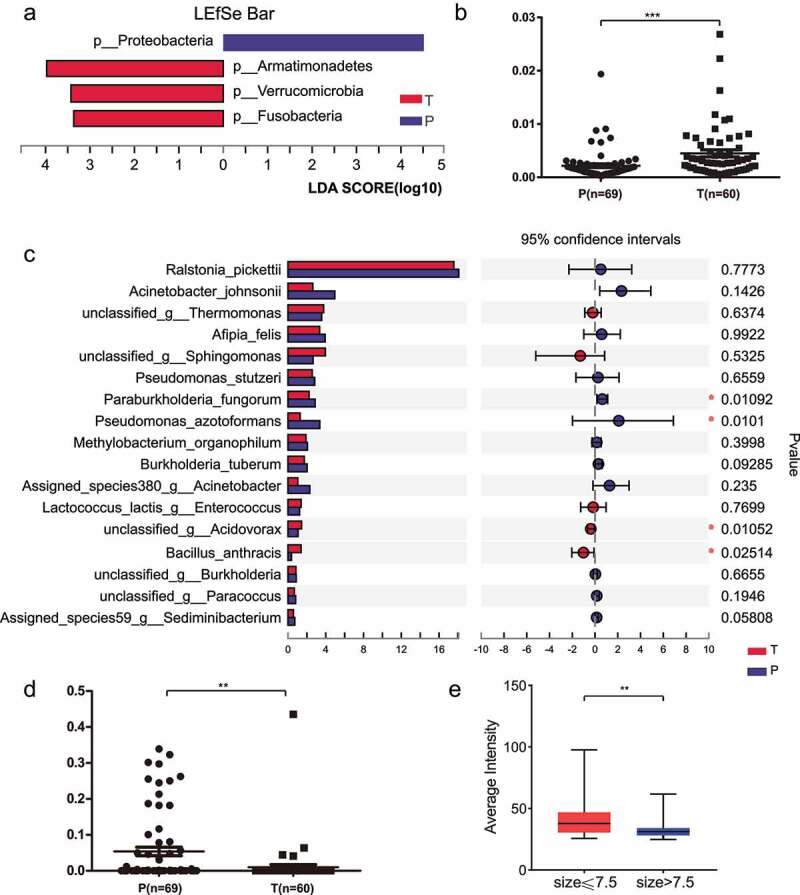
Differential analysis of bacteria content between tumor and paracancerous tissues. (a) Performance of the linear discriminant analysis (LDA) at the phylum level to estimate the magnitude of each species’ abundance effect on the differential expression. (b) qPCR validation of the differential abundance of the phylum Verrucomicrobia in a dependent cohort. (c) Species levels of the differentially expressed bacterium between tumor and paracancerous tissues. Multiple testing correction using two-tailed Wilcoxon test and FDR; *P value < .05; CI calculated by the bootstrap method using 95% CI. (d) qPCR validation for phylum Proteobacteria in a dependent cohort between tumor and paracancerous tissues. (e) The relationship between *P. fungorum* content (obtained by FISH fluorescence semi-quantitative) and tumor size (threshold set to 7.5 cm) was investigated using an independent cohort. T, tumor tissues; P, paracancerous tissues.

Considering the tissue differences between cancers and paracancers, the characteristics of their differential bacterial populations at the level of each species were calculated. At the Phylum level, linear discriminant analysis (LDA) results showed a higher abundance of Proteobacteria in paracancerous tissues, while Armatimonadetes, Verrucomicrobia and Fusobacteria were more abundant in cancerous tissues (Figure 6a). The LDA results at the genus level are shown in Supplementary Fig. S4. Overabundance of Verrucomicrobia DNA in the tumor tissues of ICC patients was further validated by an independent quantitative molecular approach (RT-qPCR) using primer sequences with high specificity (Figure 6b). Depletion of Proteobacteria DNA in the tumor tissues was further validated by qPCR (Figure 6d). At the family level, most of the differential organisms showed high levels in tumor tissues, including Comamonadaceae, Staphylococcaceae, Verrucomicrobiaceae, Thiobacteriaceae, and Peptococcaceae. At the genus level, most abundant bacteria exhibited high levels in tumor tissues, including *Acidovorax, Staphylococcus, Bdellovibrio, Roseimicrobium*, and *Roseburia*. At the species level, *Ralstonia pickettii* and *Acinetobacter johnsonii* accounted for the largest amount (Figure 6c). However, it is possible that these two species are contaminating bacteria in the environment, and they are widely distributed in drinking water, soil, and hospital environments, among others. Among them, *P. fungorum* and *P. azotoformans* were the bacteria with significant differences in cancer and paracancerous tissues (Figure 6c; Supplementary Table S4). The results showed that *P. fungorum* and *P. azotoformans* were more abundant in paracancerous tissues than in cancerous tissues. We conjecture that bacteria with higher levels in paracancerous tissues have an inhibitory effect on tumor development, similar to the results of fluorescence semi-quantitative analysis for *P. fungorum* (Figure 6e). The results of differential abundance at the family and genus levels are shown in the supplemental materials (Supplementary Fig. S5-S6).

We have computationally identified differentially characterized bacterial populations in tumor and paracancerous tissues, but the evolutionary relationships between the microbiota are unclear. To explore the relationships between bacteria in ICC tissues, we constructed a phylogenetic tree (Supplementary Fig. S7). The results showed that genera that differed relatively less from *Burkholderia* include *Ralstonia, Pelomonas*, and *Acidovorax*. The genera *Pseudomonas* and *Thermomonas* were evolutionarily more related. The genera *Afipia, Methylobacterium, Sphingomonas*, and *Paracoccus* showed higher species similarity.

### P. fungorum *inhibits bile duct cancer cell migration and proliferation*

**Figure 7.**
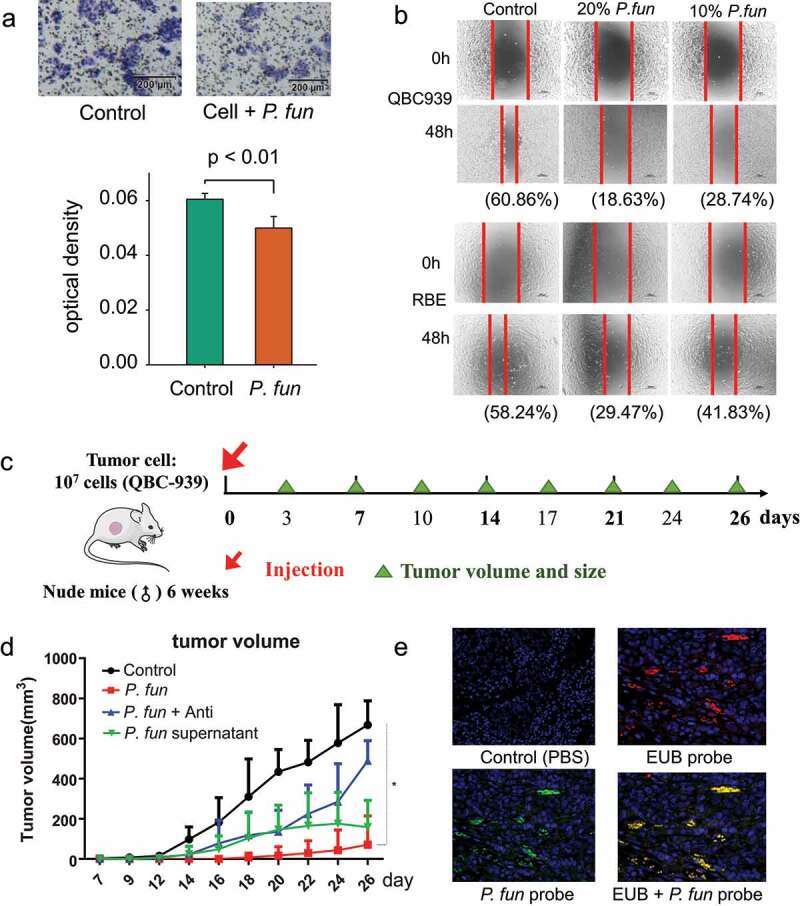
In vitro and in vivo assays show the inhibitory effect of *P. fungorum* on tumor growth. (a) Transwell assay of *P. fungorum* and bile duct cancer cells. (b) Scratch assay of QBC939 (top panel) and RBE cells (bottom panel) with the addition of bacterial *P. fungorum* supernatant (no viable bacteria). Percentages in parentheses indicate the percentage of scratch healing. (c) Schematic diagram of the mouse experiment. (d) Volume changes of transplanted tumors in PBS and *P. fungorum*-treated mice in vivo experiments. (e) FISH fluorescence staining of nude mouse transplanted tumor sections. The red color indicates the EUB probe signal, while the green color indicates the *P. fun* probe signal. *P. fun, Paraburkholderia fungorum.*

Considering that the supernatant of the bacterial fluid contains macromolecules and bacterial metabolites, experiments were designed to investigate the functions performed by these bacteria. The human cholangiocarcinoma cell lines RBE and QBC939 were utilized in our study. Methyl thiazolyl tetrazolium (MTT), transwell and scratch assays were used to study the effect of *P. fungorum* metabolites on cancer cell migration. Considering the inhibitory effect of S. aureus on hepatocellular carcinoma progression^27^ and that *S. aureus* and *S. capitis* are highly homologous,^28^ we performed in vitro and in vivo experiments using *S. capitis* obtained in culture as a positive control. The MTT assay results displayed that the supernatant of the *P. fungorum* solution (pre-filtered and devoid of viable bacteria) could inhibit cell proliferation, and the higher the concentration, the more effective the inhibition (Supplementary Fig. S8). The results of transwell assays demonstrated that the number of cells per unit area under electron microscopy was significantly reduced after the addition of *P. fungorum* supernatant, which represented a diminished proliferation and migration ability of cells (Figure 7a).

Similarly, we investigated the effect of bacterial supernatant on the migration ability of cholangiocarcinoma cells by scratch assay. The percentage of cell scratch healing was significantly lower in QBC939 cells after adding the supernatant of *P. fungorum* or *S. capitis* than in the control samples (Figure 7b; Supplementary Fig. S9A and Fig. S9B), and the scratch healing rate decreased with a gradual increase in the concentration of bacterial supernatant. A similar phenomenon was observed in the RBE cell line. Comparing the two cell lines, QBC939 had a stronger cell migration ability than the RBE cell line. Taken together, these results indicate that bacterial supernatant inhibits the migration of different types of bile duct cancer cells, which predicts the possible presence of factors in bacterial metabolites that inhibit cancer cell migration.

Next, we studied the effect of bacteria on tumor phenotypes and their possible functions in vivo experiments. We utilized the QBC939 cell line and constructed a subcutaneous transplantation tumor model in 6-week-old nude mice (Figure 7c). In addition to the conventional subcutaneous transplantation of tumor cells into mice as a control group, the other three experimental groups were designed: tumor cells plus live bacteria, tumor cells plus bacterial supernatant, and tumor cells plus live bacteria with the addition of antibiotics to the drinking water of mice to ablate bacteria in mice. The antibiotics were a mixture of penicillin and streptomycin, and we pre-tested the sensitivity of the bacteria to the antibiotics and found that the antibiotic mixture could effectively inhibit the experimental bacteria. The results of in vivo experiments showed that the tumor volume of mice in the experimental group was reduced relative to the control group (Figure 7d; Supplementary Fig. S9C). The tumor volume of mice with live bacteria in the experimental group was the lowest compared to the control group, and there was a statistically significant difference when compared to the control group. Among the experimental groups, there was no significant difference in their volume changes between the bacterial supernatant group and the antibiotic-treated group from inoculation to the 3 weeks, although the average volume of tumors was eventually larger in the antibiotic group. To demonstrate the existence of bacteria in mouse tumor tissues, we stained the tumor sections of the experimental group of mice treated with live bacteria. Using different probes, we verified the presence of P. fun in mouse tumors using FISH experiments, which also illustrates the accuracy of our in vivo experiments to some extent (Figure 7e).

### Metabolomics and transcriptomics of transplanted tumors show that bacteria can affect amino acid metabolic pathways

**Figure 8.**
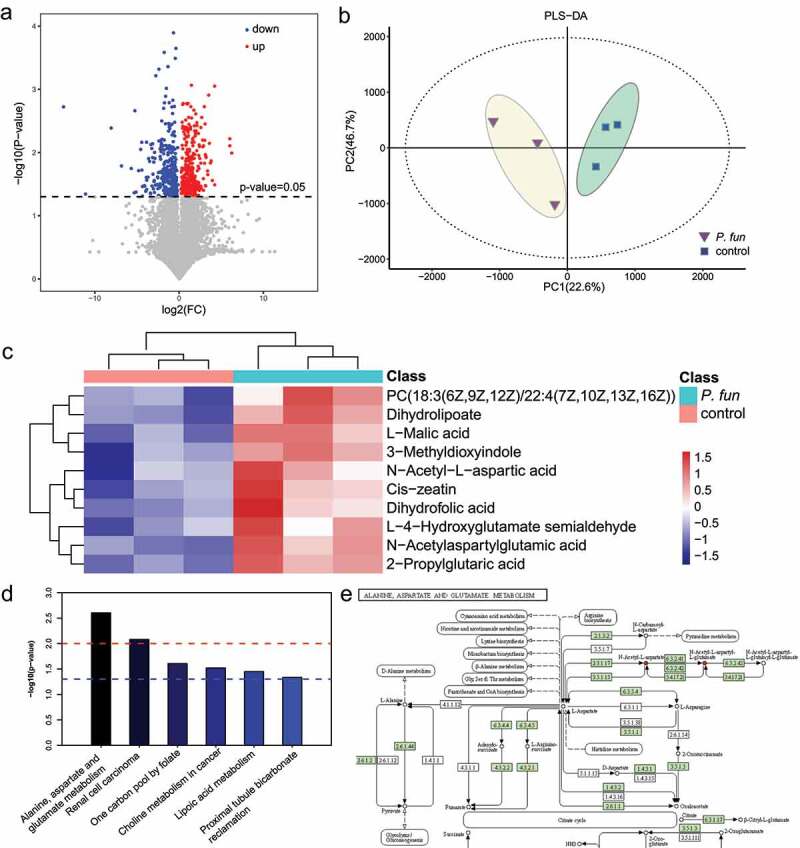
Metabolomic analysis of subcutaneous transplanted tumors in mice. (a) Volcano plot of differential metabolites. (b) Principal component analysis of metabolomics data. (c) Expression abundance of differential metabolites. The colors from blue to red indicate the metabolite expression abundance from low to high. (d) Results of pathway enrichment analysis of differential metabolites. (e) Schematic diagram of the alanine, aspartate and glutamate metabolic pathways. The red circles indicated by red arrows in the figure denote the upregulated metabolites.

To explore the effect of bacteria on tumors, samples were collected from mouse transplanted tumors and liquid chromatography-mass spectrometry (LC-MS)-based untargeted metabolomics was performed. The results of univariate statistical analysis showed that a total of 380 metabolites were significantly different (screening criteria: P value < .05 and |Fold change| >2) in tumors transplanted from experimental and control mice, of which 187 were expressed down-regulated and 193 were up-regulated (Figure 8a). The results of the principal component analysis showed that the two groups of sample data could be well distinguished (Figure 8b), indicating the existence of different biological characteristics between the two groups. By comparative analysis, after the treatment of transplanted tumors with *P. fungorum*, the metabolites 2-Propylglutaric acid, 3-Methyldioxyindole, Dihydrofolic acid, Dihydrolipoate, L-4-Hydroxyglutamate semialdehyde, N-Acetyl-L-aspartic acid and N-Acetyl-aspartyl glutamic acid showed an up-regulation trend (Figure 8c; Supplementary Table S5). The most significantly upregulated metabolites were 2-propylglutaric acid (fatty acid class, probably related to drug metabolism) and N-acetylaspartic glutamate (amino acids, peptides and their analogs). Similarly, we counted the categories of the above metabolites and found that most of the differential metabolites belonged to either fatty acids or amino acids, peptides and analogues. Next, a KEGG-based pathway enrichment analysis was performed for the differential metabolites. The results showed that the differential metabolites were mainly enriched in the metabolic pathways of alanine, aspartate and glutamate metabolism (Figure 8d; Supplementary Table S6). By combining the results of the functional prediction analysis with previous microbiome data, we could see an increased amount of N-acetyl-L-aspartate in this pathway (Figure 8e), which might be caused by bacterial action.

In addition to probing metabolite changes with metabolomics of mouse transplanted tumors, transcriptomic assays were performed using supernatant-treated cell lines to detect gene expression changes. KEGG metabolic pathway enrichment analysis revealed that differentially expressed genes were mainly enriched in carbon metabolism, glycolysis/gluconeogenesis, amino acid biosynthesis, aminoacyl-tRNA biosynthesis, pyruvate metabolism, cancer central metabolism, and carbon metabolism pathways (Supplementary Fig. S10; Supplementary Tables S7-S8). By comparing the results with the metabolomics results, we infer that amino acid anabolism is closely related to the bacterial action on tumors.

## Discussion

The presence of bacteria in human tumors has been reported for more than a hundred years, but a systematic and complete understanding of the characteristics and biological significance of bacteria is lacking. The low microbial content in tumor tissues makes it difficult to find an appropriate biological model to initiate systematic validation of them.^29,30^ Previously, researchers in Singapore studied the intratumoral microbiota of Thai cholangiocarcinoma patients, some of whom were infected with *Schistosoma hepatica*, which is very different from the clinical background of our native Chinese cholangiocarcinoma patients.^18^ As far as the research methodology is concerned, in addition to the analysis of microbiome data, we went a step further and discussed the mechanism of microbial action on tumors at the molecular level. In addition, using fresh tissue homogenate cultures, we identified and isolated live bacteria from them, which demonstrated the intracellular presence of bacteria in tumor tissues in the form of live bacteria. By setting up a negative control, we used computational biology to remove contaminating bacteria and thus more accurately characterize the microbiota in ICC tissues. Previous researchers have also used large sample sizes to demonstrate that many of the bacteria in tumor tissues are intracellular and that they are present in both cancer cells and immune cells.^17^ This predicts that there is no distance barrier between bacteria and tumor cells, which implies a direct interaction between them. Additionally, some investigators have also found that the number of bacteria in adipose tissue correlates with indicators of immune cell infiltration, inflammation and metabolism.^31^ Therefore, based on the analysis of microbiome data from clinical samples, we have considered two research directions for subsequent experimental validation, one of which is the effect of bacteria on the tumor microenvironment, and the other is the effect of bacteria on tumor tissue metabolism.

The results of the microbiome data showed that most of the highly abundant bacteria were less expressed in ICC tissues relative to paracancerous tissues. Among the differences, *P. fungorum* was higher in paracancerous tissues, and the abundance of this bacterium showed a weak negative correlation with the level of CA199, a clinical cancer antigen indicator. Therefore, we speculate that metabolites of this bacterium may suppress tumor cells. Riquelme et al. found higher levels of microorganisms in the tumors of long-term surviving patients and showed immune activation relative to short-term surviving patients with pancreatic cancer.^32^ On this basis, it can be assumed that certain bacteria play an active role in tumor suppression and that they can be directly or indirectly involved in the “anti-tumor” process. However, it has also been found that treatment of xenograft colon cancer mice with metronidazole, an antibiotic that inhibits Bacillus nuclei in colorectal cancer tissues, can inhibit the proliferation of cancer cells.^33^ In summary, we conclude that for different tumor types, diverse intratumoral bacteria have distinct effects on tumor progression.

Through in vitro and in vivo experiments, we verified that *Burkholderia* blocked tumor cell migration and significantly inhibited the growth of transplanted tumors in mice with cholangiocarcinoma. Given this, we linked a nonpathogenic *Escherichia coli* strain capable of expressing CD47nb, which was designed by Sreyan et al. at Columbia University using a synthetic biology approach, where local injection of this bacterium in mouse lymphoma tumors stimulated an anti-tumor immune response that led to complete tumor regression in mice and even control of tumor lesions distal to the uninjected strain.^34^ Thus, changes in microbial abundance can also be considered as a process by which microorganisms interact with human host cells, with alterations that have multiple effects, one of which is on the tumor immune response. It has been shown that microorganisms in pancreatic cancer can induce immune infiltration and thus prolong patient survival.^32^ In addition, metabolites of the microbial community may influence tumor development. Several studies have reported that the metabolism of human intestinal bacteria can interact with the products of host genes.^35^ Metabolites between the host and its bacterial symbionts have also been reported to control host tumor immunity.^36^ Considering these two points, we should choose immunologically intact tumor-bearing mouse models. However, due to laboratory conditions and technical limitations, we were unable to successfully construct a spontaneous tumor mouse model of ICC. Therefore, we conducted an exploratory study to solve the problem by using a xenograft tumor model, focusing on the relationship between bacteria and tumor metabolism. In addition, the inhibition of transplant tumor growth by the live bacteria was more pronounced compared to the supernatant of the bacterial solution, which may be since the live bacteria metabolize more vigorously and produce more metabolites. It is noteworthy that the size of transplanted tumors in the experimental group with antibiotics added to the drinking water was significantly reduced compared to the control group, and the change in the size of transplanted tumors in this group was similar to that in the group containing only live bacteria. The reason for this may be that the addition of antibiotics to the drinking water did not ensure that the drug reached the tumor sites of the mice rapidly.

For the functional mechanism study, through microbiome data analysis, we identified microbial features of cancer and paracancerous tissue differences and predicted their association with amino acid metabolic pathways. We performed metabolomic assays on mouse transplanted tumors. By analyzing the differences in metabolites between experimental and control groups, we infer that the colony may influence tumor progression through the biological process of amino acid metabolism.

The discussion of the origin of intratumor microbes is a very interesting issue. Riquelme and his colleagues showed cross-talks between intra-tumor microbes in resected pancreatic adenocarcinoma, and intestinal bacterial microbiota, which can modulate the host’s antitumor immune response.^32^ Other similar studies have shown that gut microbes usually regulate tumor growth through tumor immunity and that intratumoral microbe may exert an essential role,^37–39^ implying a close relationship between intratumoral and gut microbiota. In particular, due to the presence of enterohepatic circulation (EHC), some substances are excreted into the intestine via the bile, reabsorbed in the intestine, and eventually returned to the liver via the portal vein.^40,41^ Considering that this mechanism of hepatic-intestinal circulation is likely to create conditions for cross-talks between intestinal microbes and intratumoral bacteria, we postulated that ICC intratumoral microbes are also derived from intestinal microbes to a large extent. However, further experiments are needed to verify the hypothesis.

In conclusion, our findings demonstrate that bacteria in ICC may play an important antitumor role and that bacterial biomass in the tumor correlates with clinical factors. Thus, DNA of bacterial microbiota can be used as a diagnostic marker or prognostic marker for tumors, or even as a target for clinical drug therapy. However, the molecular mechanisms of how exactly they interact with cancer cells remain to be deeply explored. We need to understand clearly the molecular mechanisms of how microbiota enter and reside in tumor tissues, and study how to utilize them as targets for tumor prevention.

## Patients and methods

### Patients selection and samples collection

The samples were collected from patients diagnosed with ICC who had previously undergone curative resection surgery without any adjuvant treatment. Considering the possibility of endogenous contamination during the procedure, we tried to select clean tissue blocks for the procedure experiments. All patients were from Zhongshan Hospital, Fudan University. The study was approved by the Research Ethics Committee of Zhongshan Hospital, and written informed consent was obtained from each patient. Detailed information is available in the supplementary material.

### 16S rRNA sequencing

DNA from tissues was extracted using the same protocol as follows. Briefly speaking, tissues (30 ~ 50 mg) were ground in disposable grinding tubes (volume 2 mL) with one steel ball (diameter 0.6 cm) in each tube. The grinding tubes were pre-cooled with liquid nitrogen and the tissue was ground for 10 seconds at 50 Hz using a tissue grinder (WB2017075, Shanghai WonBio Biotechnology Co., Ltd.). DNA extraction was performed with FastDNA Spin Kit for soil (MP Biomedicals, USA, 116560200-CF) according to the instructions (https://www.mpbio.com/116560000-fastdna-spin-kit-for-soil-samp-cf). DNA concentration and purity were measured using a NanoDrop 2000 UV-Vis spectrophotometer (Thermo Scientific, Wilmington, USA) according to the manufacturer’s directions. The OD260/OD280 ratios of all DNA samples were greater than 1.9, indicating that the quality of the DNA samples was up to standard. The average DNA concentration of all samples was 160.39 ± 84.87 ng/µL. The quality of the DNA samples was further assessed using 1% agarose gel electrophoresis.

The primer pairs we used were 338 F (5’-ACTCCTACGGGAGGCAGCA-3’) and 806 R (5’-GGACTACHVGGGTWTCTAAT-3’), and the V3-V4 region of the bacterial 16s rDNA gene was amplified in a GeneAmp PCR System 9700 Thermal Cycler (Applied Biosystems Inc, CA, USA) using TransStart Fastpfu DNA Polymerase (TransGen AP221-02, TransGen Biotech Co.) to amplify the V3-V4 region of the bacterial 16s rDNA gene. To ensure the accuracy and reliability of subsequent data analysis, we used low cycle number amplification whenever possible, using the same number of cycles for each sample. The reaction system contains 4 μL 5× FastPfu buffer, 2 μL 2.5 mM dNTPs, 0.8 μL forward primer (5 μM), 0.8 μL reverse primer (5 μM), 0.4 μL FastPfu polymerase, 0.2 μL BSA and 10 ng template DNA. The volume is then adjusted to 20 μL with water. The first round of PCR reaction consists of the following steps. 95°C for 3 min; 23 cycles (95°C for 30s; 55°C for 30s; 72°C for 45s); 72°C for 10 min; and 10°C until stopped by the user. A second round of PCR was performed to increase the index, using the following procedure. 95°C, 3 min; 8 cycles (95°C for 30s; 55°C for 30s; 72°C for 45s); 72°C for 10 min; and 10°C until stopped by the user.

The amplification parameters were adjusted in preliminary experiments. PCR products were analyzed using 2% agarose gel electrophoresis. PCR products were classified as A (strong), B (moderate), and C (weak or invisible) according to their size and concentration. Samples A and B were regarded as qualified samples and sequenced. For samples with score C, DNA extraction and PCR amplification were replicated using residual tissue from the same specimen. For sequencing, three PCR experiments were conducted in parallel for each sample. PCR products from each sample were merged and subjected to 2% agarose gel electrophoresis. PCR fragments were recycled from the gels using the AxyPrep DNA Gel Extraction Kit (AXYGEN Biosciences, Union City, CA, USA). DNA was eluted using Tris-HCl buffer and its recovery and quality were evaluated by 2% agarose gel electrophoresis. DNA concentration was measured using a QuantiFluor-ST handheld fluorometer (Promega Corporation, Madison, WI, USA).

Miseq libraries for sequencing are built by adding Illumina adapters to the ends of target DNA by PCR using the TruSeq DNA Sample Preparation Kit. PCR products were retrieved from agarose gels using the AxyPrep DNA Gel Extraction Kit. We eluted the DNA with Tris-HCl buffer and evaluated its quality by 2% agarose gel electrophoresis. Double-stranded DNA was denatured with NaOH to generate single-stranded DNA. Purified amplicons were pooled in equimolar patterns and analyzed on the Illumina MiSeq PE300 platform/NovaSeq PE250 platform (Illumina, San Diego, USA) in paired-end mode.

### Analysis of 16S rRNA sequencing data

Quality control to remove low-quality reads data using fastqc software^42^ and cutadapter software.^43^ The reads sequences from double-end sequencing were merged with FLASH software.^44^ The specific QC steps are as follows: (1) filter the bases with a tail mass value below 20 in the reads, set a window of 50bp, and truncate the back-end bases from the window if the average mass value within the window is below 20; filter the reads below 50bp after QC, and remove the reads containing N bases; (2) merge pairs of reads into one sequence according to the overlapping relationship between PE reads, with a minimum overlap length of 10bp, and the maximum mismatch ratio allowed for the overlap region is 0.2; (3) distinguish samples according to the barcode and primers at the beginning and end of the sequence, and adjust the sequence orientation, the number of mismatches allowed for barcode is 0, and the maximum number of primer mismatches is 2. The clean data were processed into OTUs sequences with UPARSE software^45^ to complete OTU clustering. The 16S rRNA database Silva v138 (https://www.arb-silva.de/)^46^ was compared with the RDP Classifier software^47^ and annotated for each OTU sequence to generate OTU tables. In addition to the Silva database, we also annotated the OTU sequences with the 16S rRNA database RDP (http://rdp.cme.msu.edu/)^48^ and GreenGenes (http://greengenes.secondgenome.com/). Contaminating bacteria were removed with the Decontam program^19^ with the parameter “method = frequency”. Calculation of colony Alpha diversity using Mothur software.^49^ Qiime software^50^ was used to generate abundance tables for each taxonomic level of the microbiota and to calculate the Beta diversity of the microbiota. Phylogenetic trees were constructed and evolutionary analyses were performed using Mega software^51^ and IQ-TREE.^52^ Differential bacterial populations were identified on the Majorbio Cloud Platform using linear discriminant analysis (LEfSe, https://huttenhower.sph.harvard.edu/galaxy/).^53^ The PICRUST2 tool^54^combined with the KEGG database^55^ (https://www.genome.jp/kegg/) and the EggNOG database^56^ (https://eggnog.embl.de/) was used to predict biological functions.

### Single-cell RNA-sequencing (scRNA-seq)

Fresh tissue samples were immediately stored upon collection in the GEXSCOPE^TM^ Tissue Preservation Solution (Singleron Biotechnol-ogies) within 2–8°C. Before digested in GEXSCOPE^TM^ Tissue Dissociation Solution (Singleron Biotechnologies) at 37°C, the specimens were firstly washed with HBSS for three times and grinded into 1–2 mm pieces. After digestion, cell debris was filtered out and the cells were centrifuged at 1000 rpm, 4°C, for 5 minutes and cell pellets were resuspended into 1 ml PBS. To remove red blood cells, 2 mL GEXSCOPE^TM^ Red Blood Cell Lysis Buffer (Singleron Biotechnologies) was added to the cell suspension and incubated at 25°C for 10 minutes. The mixture was centrifuged at 1000 rpm for 5 min and the cell pellet was then resuspended in PBS. Single cell suspension was loaded onto a microfluidic chip and single cell RNA-seq libraries were constructed according to the manufacturer’s instructions (Singleron Biotechno-logies). The resulting single cell RNA-seq libraries were sequenced on an Illumina HiSeq X10 instrument with 150bp paired-end reads.

### Analysis of the scRNA-seq datasets

The data analysis of the scRNA-seq follows a conventional approach with the following simple steps. QC, filtering, comparison and quantification of raw sequencing data using the SCOPE-tools toolkit to generate a single cell matrix. Single-cell sequencing reads data were processed using Kraken2 software^57,58^ and Bracken software^59^ to obtain the abundance matrix of the bacterial population. The raw gene expression matrix was imported and processed using the R package Seurat.^60^ Data were filtered by the following criteria: the sum of expression of all genes measured per cell (nCount_RNA) was greater than 201, the number of genes measured per cell (nFeature_RNA) was more than 6,000 or less than 201, and the percentage of mitochondrial genes (percent.mt) was no more than 20%. The gene expression matrix of the remaining high-quality cells was normalized by the total number of cellular UMIs. The normalized expression was scaled by the total number of cellular UMIs and the percentage of mitochondrial genes (scale.factor = 1e4). Highly variable genes were calculated using the Seurat FindVariableGenes function with all parameters as default except select.method = “vst” and features = 2000. PCA analysis was then performed with highly variable genes, and significant principal components (top 10) were selected for dimensionality reduction, and clusters were identified using the FindClusters function (dims.use = 1:10, resolution = 0.5). Dimensionality reduction and gene expression visualization were performed using tSNE and UMAP analysis. Also, the first 10 principal components were further dimensionality reduced using the RunUMAP function with default settings. Initial annotation of cell types was performed using SingleR,^61^ and the literature on marker genes was reviewed to correct the cell annotation results.

16S rRNA sequencing, H&E staining, FISH, bacterial culture, single-cell RNA sequencing (scRNA-seq), quantitative PCR (q-PCR), in vitro and mouse experiments, and data analyses were performed, as described in the supplementary methods.

## Supplementary Material

Supplemental Material

## Data Availability

The 16S rDNA sequencing datasets have been deposited with links to BioProject accession number PRJNA753723 in the NCBI BioProject database (https://www.ncbi.nlm.nih.gov/bioproject/). The scRNA-seq data has been deposited in Gene Expression Omnibus (GEO) with accession number GSE181878. The transcriptome of QBC939 cell lines treated with bacterial supernatant has been deposited in GEO with accession number GSE182319.

## References

[1] Valle JW, Kelley RK, Nervi B, Oh DY, Zhu AX. Biliary tract cancer. England): Lancet (London; 2021. Vol. 397. p. 428–19. 10.1016/S0140-6736(21)00153-7 33516341

[2] Scheperjans F, Aho V, Pereira PA, Koskinen K, Paulin L, Pekkonen E, Haapaniemi E, Kaakkola S, Eerola‐Rautio J, Pohja M, et al. Gut microbiota are related to Parkinson’s disease and clinical phenotype. Movement Disorders: Official Journal of the Movement Disorder Society. 2015;30:350–358. doi:10.1002/mds.26069. 25476529

[3] Koeth RA, Wang Z, Levison BS, Buffa JA, Org E, Sheehy BT, Britt EB, Fu X, Wu Y, Li L, et al. Intestinal microbiota metabolism of L-carnitine, a nutrient in red meat, promotes atherosclerosis. Nat Med. 2013;19:576–585. doi:10.1038/nm.3145. 23563705 PMC3650111

[4] Chu H, Williams B, Schnabl B. Gut microbiota, fatty liver disease, and hepatocellular carcinoma. Liver Research. 2018;2:43–51. doi:10.1016/j.livres.2017.11.005. 30416839 PMC6223644

[5] Furusawa Y, Obata Y, Fukuda S, Endo TA, Nakato G, Takahashi D, Nakanishi Y, Uetake C, Kato K, Kato T, et al. Commensal microbe-derived butyrate induces the differentiation of colonic regulatory T cells. Nature. 2013;504:446–450. doi:10.1038/nature12721. 24226770

[6] Atarashi K, Tanoue T, Oshima K, Suda W, Nagano Y, Nishikawa H, Fukuda S, Saito T, Narushima S, Hase K, et al. Treg induction by a rationally selected mixture of Clostridia strains from the human microbiota. Nature. 2013;500:232–236. doi:10.1038/nature12331. 23842501

[7] Routy B, Gopalakrishnan V, Daillère R, Zitvogel L, Wargo JA, Kroemer G. The gut microbiota influences anticancer immunosurveillance and general health. Nature Reviews Clinical Oncology. 2018;15:382–396. doi:10.1038/s41571-018-0006-2. 29636538

[8] Fujimoto A, Totoki Y, Abe T, Boroevich KA, Hosoda F, Nguyen HH, Aoki M, Hosono N, Kubo M, Miya F, et al. Whole-genome sequencing of liver cancers identifies etiological influences on mutation patterns and recurrent mutations in chromatin regulators. Nature Genetics. 2012;44:760–764. doi:10.1038/ng.2291. 22634756

[9] Zhu L, Baker SS, Gill C, Liu W, Alkhouri R, Baker RD, Gill SR. Characterization of gut microbiomes in nonalcoholic steatohepatitis (NASH) patients: a connection between endogenous alcohol and NASH. Hepatology (Baltimore, Md). 2013;57:601–609. doi:10.1002/hep.26093. 23055155

[10] Chen Y, Yang F, Lu H, Wang B, Chen Y, Lei D, Wang Y, Zhu B, Li L. Characterization of fecal microbial communities in patients with liver cirrhosis. Hepatology (Baltimore, Md). 2011;54:562–572. doi:10.1002/hep.24423. 21574172

[11] Le Roy T, Llopis M, Lepage P, Bruneau A, Rabot S, Bevilacqua C, Martin P, Philippe C, Walker F, Bado A, et al. Intestinal microbiota determines development of non-alcoholic fatty liver disease in mice. Gut. 2013;62:1787–1794. doi:10.1136/gutjnl-2012-303816. 23197411

[12] Seki E, De Minicis S, Osterreicher CH, Kluwe J, Osawa Y, Brenner DA, Schwabe RF. TLR4 enhances TGF-beta signaling and hepatic fibrosis. Nat Med. 2007;13:1324–1332. doi:10.1038/nm1663. 17952090

[13] Yoshimoto S, Loo TM, Atarashi K, Kanda H, Sato S, Oyadomari S, Iwakura Y, Oshima K, Morita H, Hattori M, et al. Obesity-induced gut microbial metabolite promotes liver cancer through senescence secretome. Nature. 2013;499:97–101. doi:10.1038/nature12347. 23803760

[14] Cariati A, Puglisi R, Zaffarano R, Accarpio FT, Cetta F. *Helicobacter pylori* and the risk of benign and malignant biliary tract disease. Cancer. 2003;98:656–657. author reply 7-8. doi:10.1002/cncr.11549. 12879485

[15] Takayama S, Takahashi H, Matsuo Y, Okada Y, Takeyama H. Effect of *Helicobacter bilis* infection on human bile duct cancer cells. Digestive Diseases and Sciences. 2010;55:1905–1910. doi:10.1007/s10620-009-0946-6. 19731027

[16] Gu W, Deng X, Lee M, Sucu YD, Arevalo S, Stryke D, et al. Rapid pathogen detection by metagenomic next-generation sequencing of infected body fluids. Nat Med. 2021;27:115–124. doi:10.1038/s41591-020-1105-z. 33169017 PMC9020267

[17] Nejman D, Livyatan I, Fuks G, Gavert N, Zwang Y, Geller LT, Rotter-Maskowitz A, Weiser R, Mallel G, Gigi E, et al. The human tumor microbiome is composed of tumor type-specific intracellular bacteria. Science. 2020;368:973–980. 32467386 10.1126/science.aay9189 PMC7757858

[18] Chng KR, Chan SH, Ahq N, Li C, Jusakul A, Bertrand D, Wilm A, Choo SP, Tan DMY, Lim KH, et al. Tissue Microbiome Profiling Identifies an Enrichment of Specific Enteric Bacteria in Opisthorchis viverrini Associated Cholangiocarcinoma. EBioMedicine. 2016;8:195–202. doi:10.1016/j.ebiom.2016.04.034. 27428430 PMC4919562

[19] Davis NM, Proctor DM, Holmes SP, Relman DA, Callahan BJ. Simple statistical identification and removal of contaminant sequences in marker-gene and metagenomics data. Microbiome. 2018;6:226. doi:10.1186/s40168-018-0605-2. 30558668 PMC6298009

[20] Van der Maaten L, Hinton G. Visualizing data using t-SNE. Journal of Machine Learning Research. 2008;9 (86) :2579−2605 .

[21] Zhang M, Yang H, Wan L, Wang Z, Wang H, Ge C, Liu Y, Hao Y, Zhang D, Shi G, et al. Single-cell transcriptomic architecture and intercellular crosstalk of human intrahepatic cholangiocarcinoma. Journal of Hepatology. 2020;73:1118–1130. doi:10.1016/j.jhep.2020.05.039. 32505533

[22] Sun Y, Wu L, Zhong Y, Zhou K, Hou Y, Wang Z, Zhang Z, Xie J, Wang C, Chen D, et al. Single-cell landscape of the ecosystem in early-relapse hepatocellular carcinoma. Cell. 2021;184(2):404–21.e16. doi:10.1016/j.cell.2020.11.041. 33357445

[23] Massalha H, Bahar Halpern K, Abu-Gazala S, Jana T, Massasa EE, Moor AE, Buchauer L, Rozenberg M, Pikarsky E, Amit I. A single cell atlas of the human liver tumor microenvironment. Molecular Systems Biology. 2020;16:e9682. doi:10.15252/msb.20209682. 33332768 PMC7746227

[24] Schafflick D, Xu CA, Hartlehnert M, Cole M, Schulte-Mecklenbeck A, Lautwein T, Wolbert J, Heming M, Meuth SG, Kuhlmann T. Integrated single cell analysis of blood and cerebrospinal fluid leukocytes in multiple sclerosis. Nature Communications. 2020;11:247. doi:10.1038/s41467-019-14118-w. PMC6959356 31937773

[25] Chai X, Hu L, Zhang Y, Han W, Lu Z, Ke A, et al. Specific ACE2 expression in cholangiocytes may cause liver damage after 2019-nCoV infection. bioRxiv (2020). 2020.02.03.931766 .

[26] Kalaora S, Nagler A, Nejman D, Alon M, Barbolin C, Barnea E, Ketelaars SLC, Cheng K, Vervier K, Shental N, et al. Identification of bacteria-derived HLA-bound peptides in melanoma. Nature. 2021;592:138–143. doi:10.1038/s41586-021-03368-8. 33731925 PMC9717498

[27] Wang Z, Yu W, Qiang Y, Xu L, Ma F, Ding P, Shi L, Chang W, Mei Y, Ma X, et al. LukS-PV Inhibits Hepatocellular Carcinoma Progression by Downregulating HDAC2 Expression. Mol Ther Oncolytics. 2020;17:547–561. doi:10.1016/j.omto.2020.05.006. 32637573 PMC7321822

[28] Coates-Brown R, Moran JC, Pongchaikul P, Darby AC, Horsburgh MJ. Comparative Genomics of Staphylococcus Reveals Determinants of Speciation and Diversification of Antimicrobial Defense. Front Microbiol. 2018;9:2753. doi:10.3389/fmicb.2018.02753. 30510546 PMC6252332

[29] Neu AT, Allen EE, Roy K. Defining and quantifying the core microbiome: challenges and prospects. Proceedings of the National Academy of Sciences . 2021; 118: e2104429118. doi:10.1073/pnas.2104429118. PMC8713806 34862327

[30] Liu S, Moon CD, Zheng N, Huws S, Zhao S, Wang J. Opportunities and challenges of using metagenomic data to bring uncultured microbes into cultivation. Microbiome. 2022;10:76. doi:10.1186/s40168-022-01272-5. 35546409 PMC9097414

[31] Massier L, Chakaroun R, Tabei S, Crane A, Didt KD, Fallmann J, von Bergen M, Haange S-B, Heyne H, Stumvoll M, et al. Adipose tissue derived bacteria are associated with inflammation in obesity and type 2 diabetes. Gut. 2020;69:1796–1806. doi:10.1136/gutjnl-2019-320118. 32317332

[32] Riquelme E, Zhang Y, Zhang L, Montiel M, Zoltan M, Dong W, Quesada P, Sahin I, Chandra V, San Lucas A, et al. Tumor Microbiome Diversity and Composition Influence Pancreatic Cancer Outcomes. Cell. 2019;178:795–806.e12. doi:10.1016/j.cell.2019.07.008. 31398337 PMC7288240

[33] Bullman S, Pedamallu CS, Sicinska E, Clancy TE, Zhang X, Cai D, Neuberg D, Huang K, Guevara F, Nelson T, et al. Analysis of Fusobacterium persistence and antibiotic response in colorectal cancer. Science. 2017;358:1443–1448. doi:10.1126/science.aal5240. 29170280 PMC5823247

[34] Chowdhury S, Castro S, Coker C, Hinchliffe TE, Arpaia N, Danino T. Programmable bacteria induce durable tumor regression and systemic antitumor immunity. Nat Med. 2019;25:1057–1063. doi:10.1038/s41591-019-0498-z. 31270504 PMC6688650

[35] Postler TS, Ghosh S. Understanding the Holobiont: how Microbial Metabolites Affect Human Health and Shape the Immune System. Cell Metab. 2017;26:110–130. doi:10.1016/j.cmet.2017.05.008. 28625867 PMC5535818

[36] Song X, Sun X, Oh SF, Wu M, Zhang Y, Zheng W, Geva-Zatorsky N, Jupp R, Mathis D, Benoist C, et al. Microbial bile acid metabolites modulate gut RORγ(+) regulatory T cell homeostasis. Nature. 2020;577:410–415. doi:10.1038/s41586-019-1865-0. 31875848 PMC7274525

[37] Zitvogel L, Ayyoub M, Routy B, Kroemer G. Microbiome and Anticancer Immunosurveillance. Cell. 2016;165:276–287. doi:10.1016/j.cell.2016.03.001. 27058662

[38] Garrett WS. Cancer and the microbiota. Science. 2015;348:80–86. doi:10.1126/science.aaa4972. 25838377 PMC5535753

[39] Sepich-Poore GD, Zitvogel L, Straussman R, Hasty J, Wargo JA, Knight R. The microbiome and human cancer. Science . 2021;371: eabc4552. doi:10.1073/pnas.2104429118. PMC8767999 33766858

[40] Cai JS, Chen JH. The mechanism of enterohepatic circulation in the formation of gallstone disease. J Membr Biol. 2014;247:1067–1082. doi:10.1007/s00232-014-9715-3. 25107305 PMC4207937

[41] Chiang JYL, Ferrell JM. Bile Acid Metabolism in Liver Pathobiology. Gene Expr. 2018;18:71–87. doi:10.3727/105221618X15156018385515. 29325602 PMC5954621

[42] Wingett SW, Andrews S. FastQ Screen: a tool for multi-genome mapping and quality control. F1000Research. 2018;7:1338. doi:10.12688/f1000research.15931.1. 30254741 PMC6124377

[43] Martin M. Martin M.Cut adapt removes adapter sequences from high-throughput sequencing reads. EMBnet. EMBnet. 2011;17:10–12. doi:10.14806/ej.17.1.200.

[44] Magoč T, Salzberg SL. FLASH: fast length adjustment of short reads to improve genome assemblies. Bioinformatics. 2011;27:2957–2963. doi:10.1093/bioinformatics/btr507. 21903629 PMC3198573

[45] Edgar RC. UPARSE: highly accurate OTU sequences from microbial amplicon reads. Nature Methods. 2013;10:996–998. doi:10.1038/nmeth.2604. 23955772

[46] Quast C, Pruesse E, Yilmaz P, Gerken J, Schweer T, Yarza P, Peplies J, Glöckner FO. The SILVA ribosomal RNA gene database project: improved data processing and web-based tools. Nucleic Acids Res. 2013;41:D590–6. doi:10.1093/nar/gks1219. 23193283 PMC3531112

[47] Wang Q, Garrity GM, Tiedje JM, Cole JR. Naive Bayesian classifier for rapid assignment of rRNA sequences into the new bacterial taxonomy. Applied and Environmental Microbiology. 2007;73:5261–5267. doi:10.1128/AEM.00062-07. 17586664 PMC1950982

[48] Cole JR, Wang Q, Fish JA, Chai B, McGarrell DM, Sun Y, Brown CT, Porras-Alfaro A, Kuske CR, Tiedje JM, et al. Ribosomal Database Project: data and tools for high throughput rRNA analysis. Nucleic Acids Res. 2014;42:D633–42. doi:10.1093/nar/gkt1244. 24288368 PMC3965039

[49] Schloss PD, Westcott SL, Ryabin T, Hall JR, Hartmann M, Hollister EB, Lesniewski RA, Oakley BB, Parks DH, Robinson CJ, et al. Introducing mothur: open-source, platform-independent, community-supported software for describing and comparing microbial communities. Applied and Environmental Microbiology. 2009;75:7537–7541. doi:10.1128/AEM.01541-09. 19801464 PMC2786419

[50] Caporaso JG, Kuczynski J, Stombaugh J, Bittinger K, Bushman FD, Costello EK, Fierer N, Peña AG, Goodrich JK, Gordon JI, et al. QIIME allows analysis of high-throughput community sequencing data. Nature Methods. 2010;7:335–336. doi:10.1038/nmeth.f.303. 20383131 PMC3156573

[51] Kumar S, Stecher G, Tamura K. MEGA7: molecular Evolutionary Genetics Analysis Version 7.0 for Bigger Datasets. Molecular Biology and Evolution. 2016;33:1870–1874. doi:10.1093/molbev/msw054. 27004904 PMC8210823

[52] Nguyen LT, Schmidt HA, von Haeseler A, Minh BQ. IQ-TREE: a fast and effective stochastic algorithm for estimating maximum-likelihood phylogenies. Molecular Biology and Evolution. 2015;32:268–274. doi:10.1093/molbev/msu300. 25371430 PMC4271533

[53] Segata N, Izard J, Waldron L, Gevers D, Miropolsky L, Garrett WS, Huttenhower C. Metagenomic biomarker discovery and explanation. Genome Biology. 2011;12:R60. doi:10.1186/gb-2011-12-6-r60. 21702898 PMC3218848

[54] Douglas GM, Maffei VJ, Zaneveld JR, Yurgel SN, Brown JR, Taylor CM, Huttenhower C, Langille MGI. PICRUSt2 for prediction of metagenome functions. <jtl>nature Biotechnology. 2020;38:685–688. doi:10.1038/s41587-020-0548-6. PMC7365738 32483366

[55] Kanehisa M, Furumichi M, Tanabe M, Sato Y, Morishima K. KEGG: new perspectives on genomes, pathways, diseases and drugs. <jtl>nucleic Acids Res. 2017;45:D353–d61. doi:10.1093/nar/gkw1092. PMC5210567 27899662

[56] Huerta-Cepas J, Szklarczyk D, Heller D, Hernández-Plaza A, Forslund SK, Cook H, Mende DR, Letunic I, Rattei T, Jensen L, et al. eggNOG 5.0: a hierarchical, functionally and phylogenetically annotated orthology resource based on 5090 organisms and 2502 viruses. <jtl>nucleic Acids Res. 2019;47:D309–d14. doi:10.1093/nar/gky1085. PMC6324079 30418610

[57] Lu J, Salzberg SL. Ultrafast and accurate 16S rRNA microbial community analysis using Kraken 2. <jtl>Microbiome. 2020;8:124. doi:10.1186/s40168-020-00900-2. PMC7455996 32859275

[58] Wood DE, Lu J, Langmead B. Improved metagenomic analysis with Kraken 2. Genome Biol. 2019;20:257. doi:10.1186/s13059-019-1891-0. 31779668 PMC6883579

[59] Lu J, Breitwieser FP, Thielen P, Salzberg SL. Bracken: estimating species abundance in metagenomics data. <jtl>peerj Computer Science. 2017;3:e104. doi:10.7717/peerj-cs.104. PMC12016282 40271438

[60] Stuart T, Butler A, Hoffman P, Hafemeister C, Papalexi E, Mauck WM 3rd, Satija, R. Comprehensive integration of single-cell data. cell. 2019;177:1888–902.e21. doi:10.1016/j.cell.2019.05.031. 31178118 PMC6687398

[61] Aran D, Looney AP, Liu L, Wu E, Fong V, Hsu A, Bhattacharya, M. Reference-based analysis of lung single-cell sequencing reveals a transitional profibrotic macrophage. nature Immunology. 2019;20:163–172. doi:10.1038/s41590-018-0276-y. 30643263 PMC6340744

