# Supplementary material for: Intratumor microbiome features reveal antitumor potentials of intrahepatic cholangiocarcinoma
Source: Gut Microbes. 2022 Dec 23;15(1):2156255. doi: 10.1080/19490976.2022.2156255 (PMC9794006; doi:10.1080/19490976.2022.2156255)
Supplement: Supplemental Material [file KGMI_A_2156255_SM1310.zip › Supplementary Materials.pdf]

Intratumor microbiome features reveal antitumor potentials of intrahepatic cholangiocarcinoma

Xiaoqiang Chai*, Jie Wang*, Huanping Li*, Chao Gao*, Shuangqi Li, Chuanyuan Wei, Jianhang Huang, Yingming Tian, Jian Yuan, Jiacheng Lu, Dongmei Gao, Yimin Zheng, Cheng Huang, Jian Zhou, Guoming Shi, Aiwu Ke#, Feng Liu#, Jiabin Cai#, Jia Fan#.

Table of contents

Supplementary materials and methods 2

Supplementary Figures 12

Fig. S1. 12

Fig. S2. 13

Fig. S3. 14

Fig. S4 15

Fig. S5 16

Fig. S6 17

Fig. S7 18

Fig. S8 19

Fig. S9 20

Fig. S10 21

References 22

Supplementary materials and methods

Patients and samples collection

The main inclusion criteria for ICC patients included: the patient was diagnosed with ICC for the first time in China and the surgery was performed between June 1, 2019 and December 1, 2019 without specificity screening. All the patients were from Zhongshan Hospital of Fudan University. In this study, normal liver tissues (N) were derived from five patients with non-neoplastic diseases such as liver abscesses. The obtained clinical samples were processed with tissue preservation solution for timely preservation and used for 16S rDNA extraction and sequencing (see supplementary methods). The study was approved by the Research Ethics Committee of Zhongshan Hospital, and written informed consent was gained from every patient.

Experimental mice

The SPF-grade nude mice of BALB/C background used in this study were purchased from Beijing Vital River. All mice were housed in the SPF animal breeding room at the Department of Experimental Animals, Fudan University.

Cell lines

The human cholangiocarcinoma cell lines QBC939 and RBE used in this study were donated by the Institute of Liver Cancer, Zhongshan Hospital, Fudan University.

Bacterial purchase

The bacterial strain involved in this study was conserved under the number: CCTCC AB 2017087,

with the Latin name: *Burkholderia fungorμm* (recently renamed to *Paraburkholderia fungorum*). It is a non-model strain and was collected at Sun Yat-sen University on April 24, 2017. The strain is mainly used for taxonomic and scientific teaching, and its characteristics are mainly manifested as moist, smooth colonies with gray color.

Tumor cell culture, resuscitation, passaging and cryopreservation

We used the traditional experimental method for cell culture, recovery, passaging and lyophilization. The complete culture medium was pre-warmed. Then the frozen cells in the liquid nitrogen were thawed by rapid shaking in a 37°C water bath. Then the cell suspension was transferred to a 15 ml centrifuge tube. Next, the pre-warmed medium was added and centrifuged at 800 r for 5 min. The cell precipitate with the fresh medium was resuspended and transferred to a cell culture dish. The culture conditions were set to 37°C and 5% CO_2.

For cell passaging, PBS was first pre-warmed in advance and used to rinse the cells. When the cell density reaches 80%, aspirate off the supernatant of the adherent cells and rinse the cells twice with PBS. Add 1ml digestion solution (0.25% Trypsin-0.53mM EDTA) to the culture flask and place in a 37°C incubator. Observe the cells and terminate the digestion by adding medium in the ultra-clean table when the cells are detached. The cells were completely dislodged by gentle blowing and then centrifuged at 900r for 4 min. Resuspend the cells with the fresh and pre-warmed medium. Divide into new culture flasks in the appropriate proportions and return to the incubator.

hematoxylin-eosin (HE) staining

The clinical sample tissues were washed several times with PBS. Then the tissues were immediately

immersed in a tissue fixative containing 4% at room temperature overnight. Sections were then embedded in paraffin and obtained in sufficient volume. Paraffin sections were immersed in dimethylbenzene for 15 min. The sections were then immersed in two sets of 95% ethanol for 1 min. After hematoxylin staining for 10 min, the sections were washed in distilled water for 1 min and fractionated in 1% HCL for 2 sec. Rinse with water for 20 sec and reblue in saturated Li2CO3 aqueous solution for 15 sec. Rinse with water for 20 sec and observe under the microscope. Soak in 80%, 90%, 95%, 100% ethanol for 5 seconds, methyl salicylate for 2 minutes in sequence. dimethylbenzene I for 2 minutes, dimethylbenzene II for 2 minutes. The neutral resin was used to seal the film and nail polish was fixed.

Fluorescence in situ hybridization (FISH)

Formalin-fixed paraffin-embedded (FFPE) tissue sections were deparaffinized and rehydrated. Soak in 100% xylene for 15 minutes (twice), in 100% ethanol for 5 minutes (twice), in 85% ethanol for 5 minutes, and in 75% ethanol for 5 minutes. The sections were washed with diethyl pyrocarbonate (DEPC)-treated water. Incubate the sections in boiling citrate-EDTA antigen retrieval solution (Solarbio, No. C1010) for 10 to 15 minutes and allow the solution to cool naturally. Incubate the sections in a pre-hybridization solution at 37˚C for 1 hour. The composition of the pre-hybridization solution consists of 50% deionized formamide, 5% 50× Denhardt's solution, 5% tRNA (20 mg/ml), 15% DPEC water, 10% dextran sulfate, and 15% prepared buffer salt solution (3 M NaCl, 100 mM Tris-HCl, pH 8.0, 50 mM EDTA, 100 mM sodium phosphate). Use Cy3-labeled probe EUB338 5'-GCTGCCTCCCGTAGGAGT-3' and Cy5-labeled non-specific complement probe 5'-CGACGGAGGGCATCCTCA-3' (5 ng/μL) overnight at 37˚C as described by Nejman D., et al [1]. Wash

with 2× saline sodium citrate (SSC) at 37˚C for 10 min, 1× SSC at 37˚C for 5 min (two changes), and 0.5× SSC at room temperature for 10 min. Counterstain cell nuclei with DAPI (2 μg/μL) for 8 min in the dark. Mount the sections with fade-resistant Mountant. The signal was captured using a PANNORAMIC MIDI digital slide scanner (3DHISTECH Ltd., Budapest, Hungary). View images with CaseViewer version 2.4 (3DHISTECH Ltd. Budapest, Hungary).

The probe sequences used in the FISH experiments were as follows: Positive control, *EUB338* 5'-GCTGCCTCCCGTAGGAGT-3'; Non-specific complement probe 5'-CGACGGAGGGCATCCTCA-3'; *P. fungorum*, 5'-GGATTTCACATCTGTCTTAGCGGAC-3'; *S. capitis*, 5'-GAAGCAAGCTTCTCGTCCG-3'; *K. pneumoniae* 5'-GACAAGGTTATTAACCTCACC-3'; *P. azotoformans*, 5'-GTGTCAGTATTAGTCCAGGTGGTCG-3'.

Bacterial cultivation using fresh tissues from ICC patients

The tissue pieces removed from the animals were soaked in saline immediately, the blades were wiped with 75% alcohol, and then sterilized with the flame of an alcohol lamp. When cooled, a small piece of the tissue was cut off and used for culture. Divide the cut tissue in two and put them into 2mL EP tubes already filled with BHI and GAM medium. Place an autoclaved stainless steel grinding bead in the tube. Place in a grinder and grind at 2500rpm, 250s, 4°C. Grind well. Then add 150 μL of the ground homogenate to each plate to coat the plate. The positive control we selected was a cotton swab to take the bacteria from the door handle in the corresponding culture solution, and the negative control was the culture solution. Aerobic culture was then placed directly in a 36°C incubator. Anaerobic culture is first put into 7.5 L anaerobic culture sealed box, and in the box into three 2.5 L anaerobic long-term package, and oxygen indicator. Then placed in 37 °C incubator incubation.

Transmission Electron Microscope (TEM)

Clinical samples were processed and tissue blocks less than 1 mm3 in volume were taken. Fix the tumor tissue with 2.5% glutaraldehyde in PBS configuration for 2 h. Rinse three times with 0.1 M PBS for 15 s each time. at the end of rinsing, add 1% osmium fixative for 2-3 h. Add 0.1 M phosphate buffer for 15 s each time and rinse three times. Then add 50% ethanol and leave it for the 20s at 4°C. Discard 50% ethanol, add 70% ethanol and leave it for the 20s at 4°C. Discard 70% ethanol, add 90% ethanol and leave it for the 20s at 4°C. Discard 90% ethanol, add 90% ethanol: acetone mixture with a volume ratio of 1:1 and leave it for the 20s at 4°C. At room temperature, add 100% acetone and repeat three times. Mix acetone and embedding solution at 2:1 and leave at room temperature for 4 h. Then mix acetone and embedding solution at 1:2 and leave at room temperature overnight. The tissue was placed in an oven at 37°C overnight, removed and transferred to an oven at 45°C for 12 h. The oven temperature was then adjusted to 60°C and placed for 24 h. The sections were sliced with a Leica UC7 ultrathin sectioning machine at a thickness of 50-60 nm and stained with 3% uranyl acetate-lecithin double lead. After staining, pictures were observed with transmission electron microscopy (FEI CM120).

Quantitative PCR (q-PCR)

In short, qPCR was performed in a Roche LightCycler® 480 II instrument (Roche Diagnostics Corporation, Indianapolis, USA) using FastStart Universal SYBR Green Master (Rox) (Roche Diagnostics GmbH, Mannheim, Germany). The following primers were used in qPCR: Ver1165F (5’-TCAKGTCAGTATGGCCCTTAT-3’) and Ver1263R (5’-CAGTTTTYAGGATTTCCTCCGCC-3’) [2]. The TestPrime 1.0 program (https://www.arb-silva.de/search/testprime/) evaluates the specificity of

the primer pair on the SILVA database. Simultaneously, we used the forward TGTTGCATGAGAAAACGCCA and reverse GTCGCCTGTTCAACCAAGGAT primer pairs [3] for qPCR analysis of human albumin exon 12. The PCR reaction system contains 2 μL of diluted DNA template (100 ng/μL), 1 μL of each primer (final concentration 1 nmol), 5 μL of 2 × FastStart Universal SYBR Green Master (Rox) mixture and 1 μL of H_2O. The qPCR amplification cycle consists of a pre-denaturation step of 95°C for 10 minutes, followed by 42 cycles of 95°C for 10 seconds and 60°C for 30 seconds. The set parameters are 95°C for 15 seconds, 60°C for 1 minute, 95°C for 10 seconds, and cooling at 40°C for 2 minutes to generate melting curves. The Delta Ct (ΔCt) method is used to standardize the expression of 16s rRNA genes (Ct (16s rRNA) – Ct (albumin)). The ΔCt value is log2 converted, and the p value is calculated using a two-sided unpaired Student's t test.

Methyl thiazolyl tetrazolium (MTT) assay

Trypsin-treated cells were counted and adjusted to the desired density. The cell suspensions were seeded in 96-well plates according to 1000cells/well and placed in a 37°C, 5% incubator. Remove one 96-well plate with cells at 24h, 48h, 72h, 96h and 120h, respectively, and add MTT solution (5mg/mL) at 20μL/well. After incubation for 4 h, discard the medium and add DMSO at 150 μL/well. shake the plate on a shaker at an appropriate speed for 10 min to fully dissolve the crystals in the cells. The OD value at 490 nm was measured, and the cell growth curve was plotted according to the results.

Transwell migration assay

The dispensed Matrigel was taken out of the refrigerator and put into the ice box at 4°C and allowed to melt slowly. And the melted Matrigel was spread onto the top layer of transwell inserts (24 wells, 8 μm) (Matrigel was diluted with serum-free RPM1640 medium at a ratio of 1:1 and 70 μL was spread

per well) and placed in a 37°C incubator to solidify overnight. The cells were treated, digested with the appropriate amount of trypsin, and resuspended by centrifugation with the appropriate amount of pre-warmed PBS. The resuspension should be serum-free medium and inoculated in the upper inserts according to 2.5 x 105 cells/well. 500 μL of cell medium containing 20% fetal bovine serum was added to the inserts and transferred to a 24-well plate, noting that After 24-48 h, discard the medium and gently wipe off any cells that have not migrated with a cotton ball. Remove the Transwell inserts, turn them upside down, and air dry. Then aspirate 500 μL of crystal violet solution (0.1%) and add it to the 24-well plate while putting in inserts, the membrane must be soaked inside the staining solution. After 15-20 min at room temperature, take out the inserts, wash them with PBS and wipe the staining solution from the upper layer and edges of the inserts with a cotton swab, and invert it. After the inserts were air-dried, five fields of view (upper left, lower left, upper right, lower right and middle) were selected under the microscope for photographing and the images were processed with ImageJ software [ref]. Finally, a 24-well plate was added with 500 μL of 33% acetic acid, and inserts were placed in this well plate, ensuring that the membrane must be completely saturated, and shaken for 10 minutes. The inserts were then removed and the OD value was measured at 570 nm on an enzyme marker, a method that indicates the number of cells that have crossed the inserts due to migration.

Wound healing assay

The cells were cultured and washed once with PBS under aseptic conditions after the density reached 80%. Add 1ml trypsin to a 10cm culture dish and place it in the incubator at 37°C for 3-5 min. Add 5ml medium to terminate the digestion, centrifuge at 800r for 5 min, and resuspend the cells with the medium. Adjust the cell density to 4E5 cells/ml for QBC939 and 1.6E5 cells/ml for RBE. Place the

culture insert in a six-well plate with sterilized forceps. Add 50ul of the cell suspension to the inserts, then add 2.8ml of the cell suspension to the 6-well plate and incubate at 37°C in a 5% CO2 incubator for 24 h. Carefully clip out the insert with sterilized forceps and add the complete culture medium and different concentrations of supernatant to the corresponding wells. The images were recorded at 0h, 24 and 48h, respectively, and converted into numerical values using ImageJ software to calculate the area of the scratch and the percentage of healing.

Mouse experiments

The QBC-939 cells were cultured in a constant temperature incubator. When the cell density reached 80%, the cells were digested with a certain amount of trypsin. Blow the serum-containing medium and trypsin together fully into individual cells, aspirate 10μL of suspension and mix well with 10μL Trypan Blue and transfer 10μL to a cell counting plate, then count the cell concentration with the instrument. Each nude mouse was inoculated with about $1x10^7$ cells under the skin with QBC-939. Using this number as a standard, the number of cells to be injected per nude mouse was resuspended into 100 μL of pre-chilled PBS. Place the approximately 6-week-old nude mice that require subcutaneous inoculation with the back facing the operator, exposing enough epidermis for injection observation. Mix the cell suspension thoroughly, aspirate 100 μL with a 1 mL syringe of 0.45 μm size, hold the mouse with the left hand so that the back side is facing the operator, scrub the epidermal area of the mouse to be inoculated with an alcohol cotton ball, and then insert the syringe needle diagonally downward at 30 degrees into the area between the epidermis and dermis of the mouse and slowly push the cell suspension in. The subcutaneously inoculated mice with QBC-939 were kept in the SPF feeding room for about three weeks, during which the growth of subcutaneous tumors was observed and recorded, the length and width of the transplanted tumors were measured, the volume of the

transplanted tumors was calculated, and the growth of the mice was observed regularly.

Metabonomics of subcutaneous xenografts

The sample preparation and the machine are following the company's protocol. After pre-cooled in a refrigerator at -20°C for 2 min, the tissues were placed in a grinder to grind (60 Hz, 2 min). Then ultrasonic extraction was performed in an ice water bath for 10 min, and then it was allowed to stand at -20°C for 30 min. Centrifuge for 10 min (13000 rpm, 4°C), and take 300 μL of the supernatant into the LC-MS vial and evaporate to dryness. Reconstitute with 300 μL methanol-water (vortex for 30 s, ultrasound for 3 min). Let stand for 2 hours at -20°C. Centrifuge for 10 min (13000 rpm, 4°C), draw 150 μL of supernatant with a syringe, filter with a 0.22 μm organic phase pinhole filter, transfer to an LC injection vial, and store at -80°C until LC-MS (AB ExionLC, AB TripleTOF 6600 plus) analysis. The chromatographic parameters are as follows: Column: ACQUITY UPLC HSS T3 (100 mm×2.1 mm, 1.8 um); column temperature: 45 ℃; mobile phase: A-water (containing 0.1% formic acid), B-acetonitrile (containing 0.1% formic acid); Flow rate: 0.35 mL/min; Injection volume: 2 μL. The subsequent statistical analysis of data is done on R.

Statistics

We used R software (version 3.6.2) for statistical analyses. For differential expression analysis, statistical significance was calculated using a two-sided Student's t-test. All measured values were expressed as mean ± SE. P value or corrected p-value < 0.05 is considered statistically significant. Multivariate statistical analyses (PCA, PLS-DA, etc.) were performed using the ropls package and plotted with pheatmap, ggplot2 and ggrepel. Correlation analysis was performed using corrplot, and

univariate statistical analysis was performed using the base package status. The Benjamini–Hochberg (BH) correction method was used for the correlation analysis.

Supplementary Figures

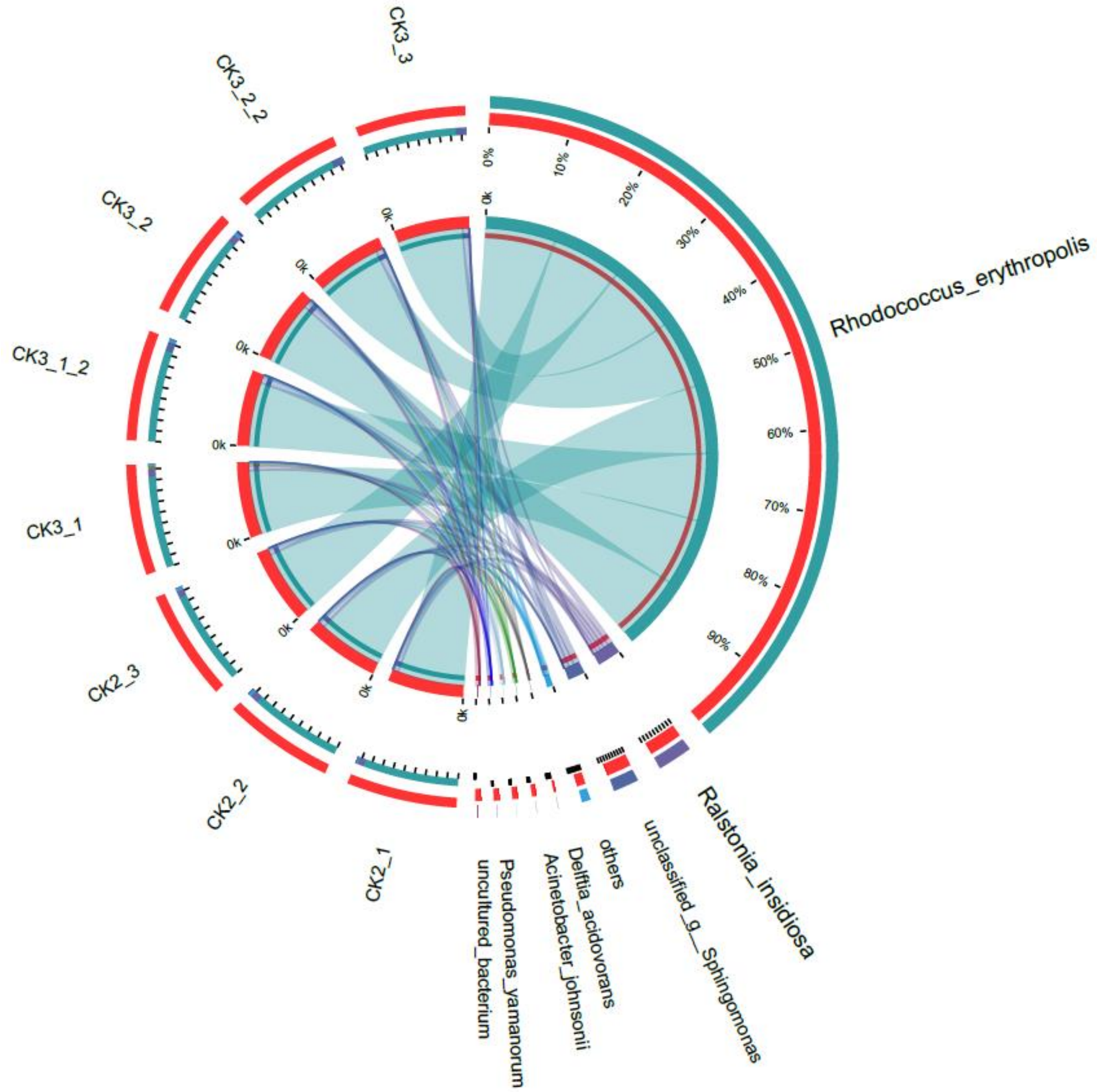

Fig. S1. Circos plot of bacterial composition in the negative controls (DNA extraction and PCR process).

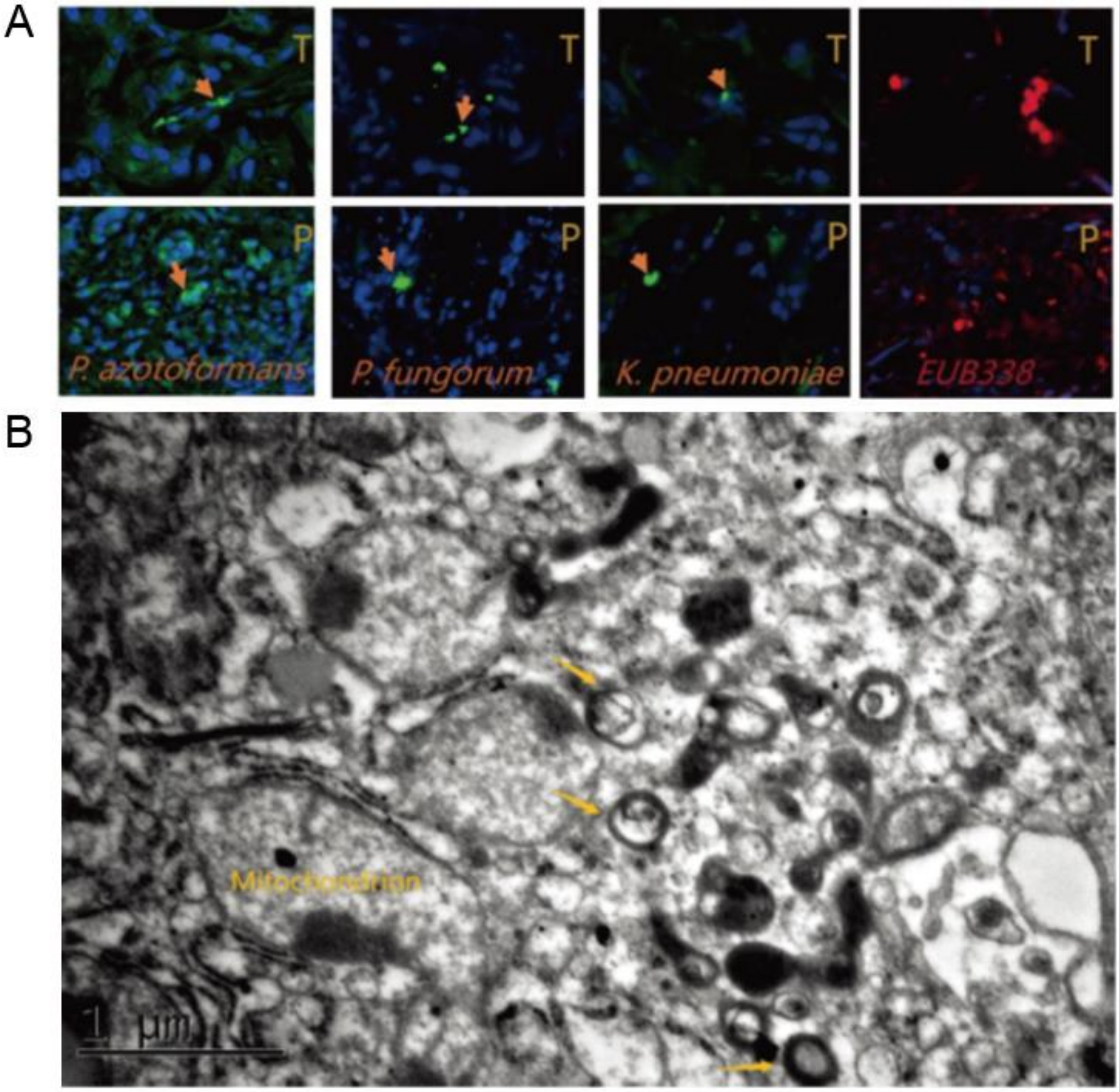

Fig. S2. Experiment to verify the existence of bacteria in ICC. (A) FISH experiment for specific bacterial species. EUB338, a probe complementary to a 16s rRNA region conserved for all bacteria. (B) Transmission electron microscopy results of bacteria in paracancerous tissues.

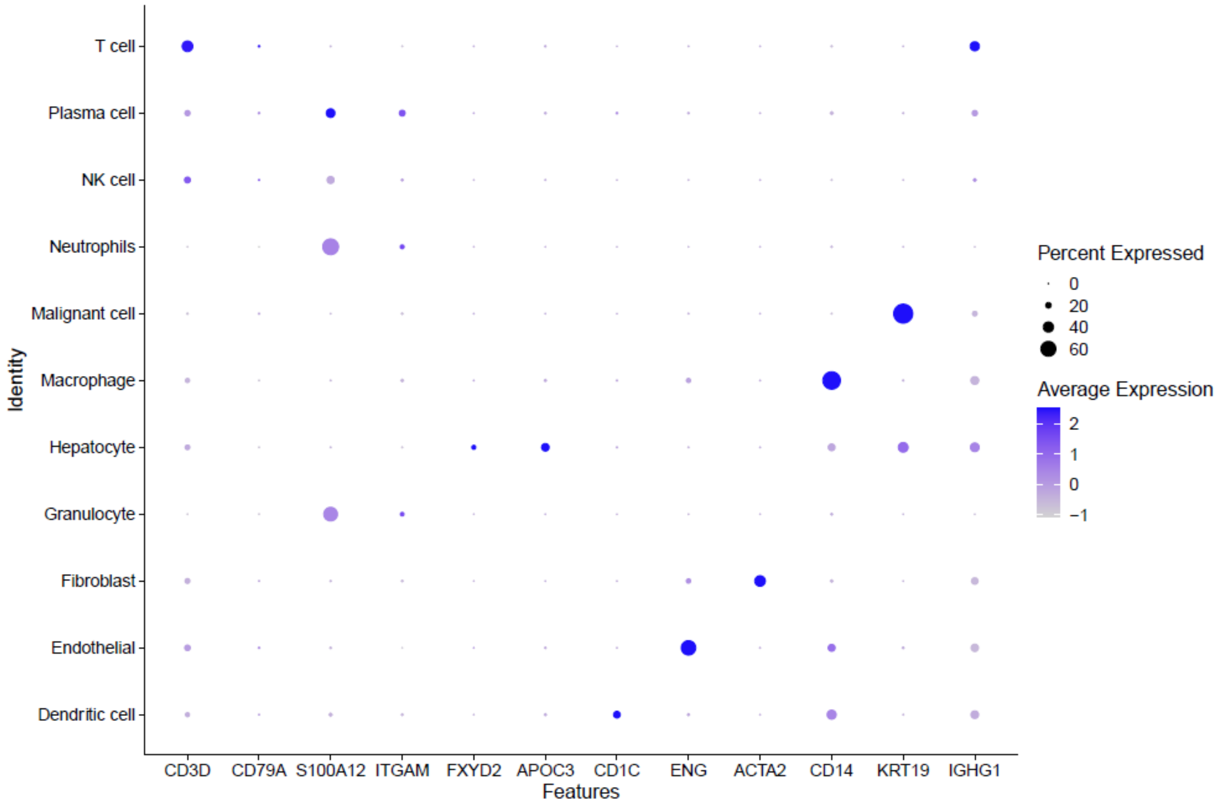

Fig. S3. Presentation of marker genes in our single-cell RNA sequencing data analysis.

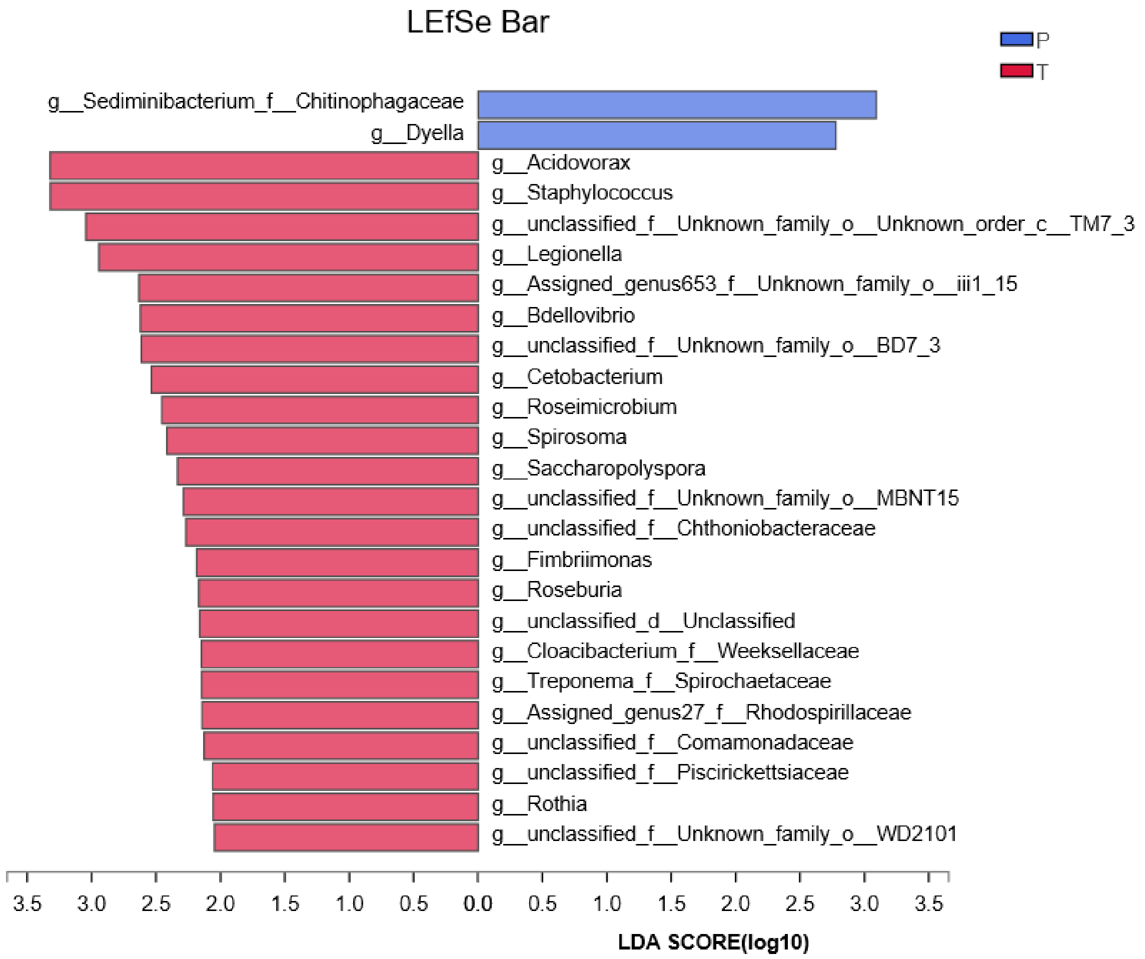

Fig. S4. At the genus level, LDA was performed to estimate the magnitude of the effect of each species' abundance on the differential effect.

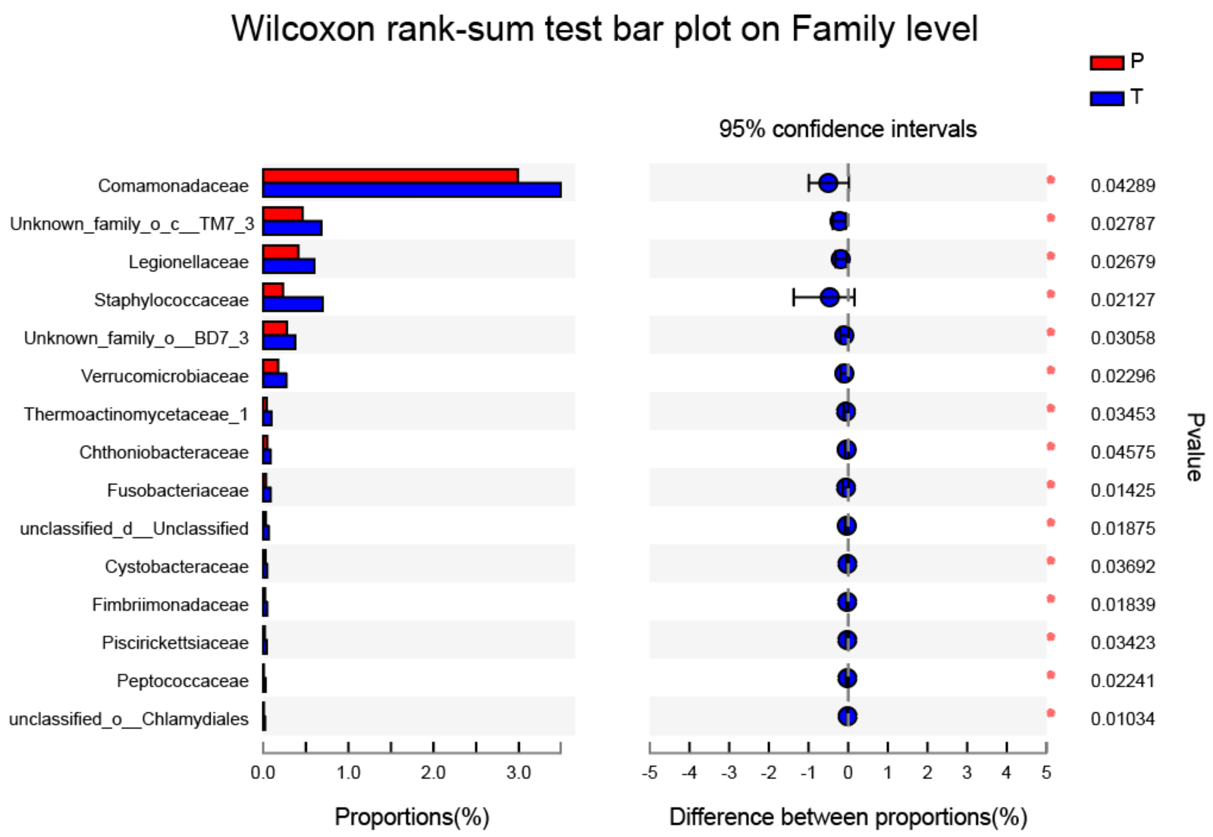

Fig. S5. Bacteria with differential content of in cancer and paracancerous tissues at the Family level.

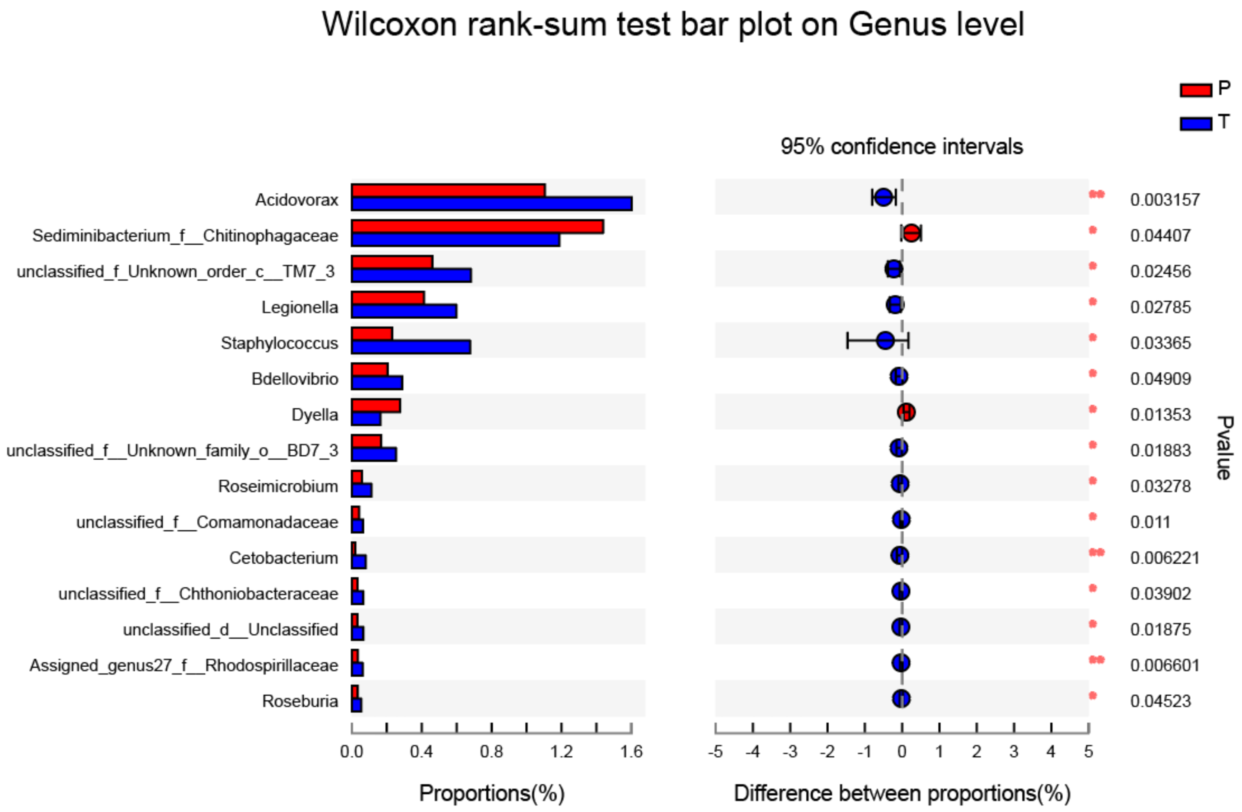

Fig. S6. Bacteria with differential content of in cancer and paracancerous tissues at the Genus level.

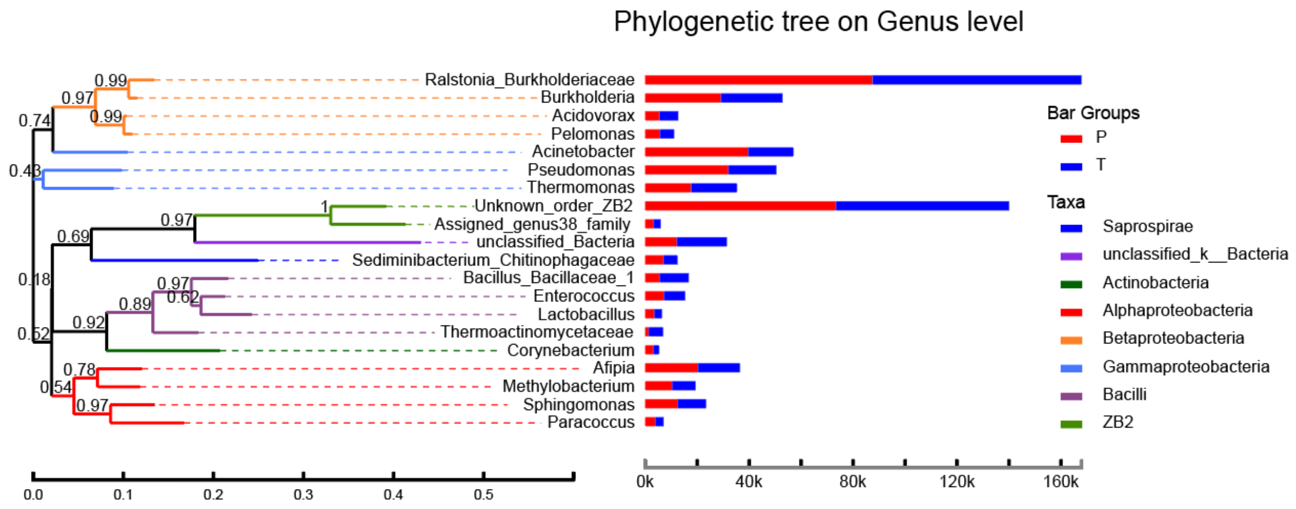

Fig. S7. Phylogenetic tree at the genus level of the intratumoral bacterium. The degree of variation of species on the same branch is much smaller.

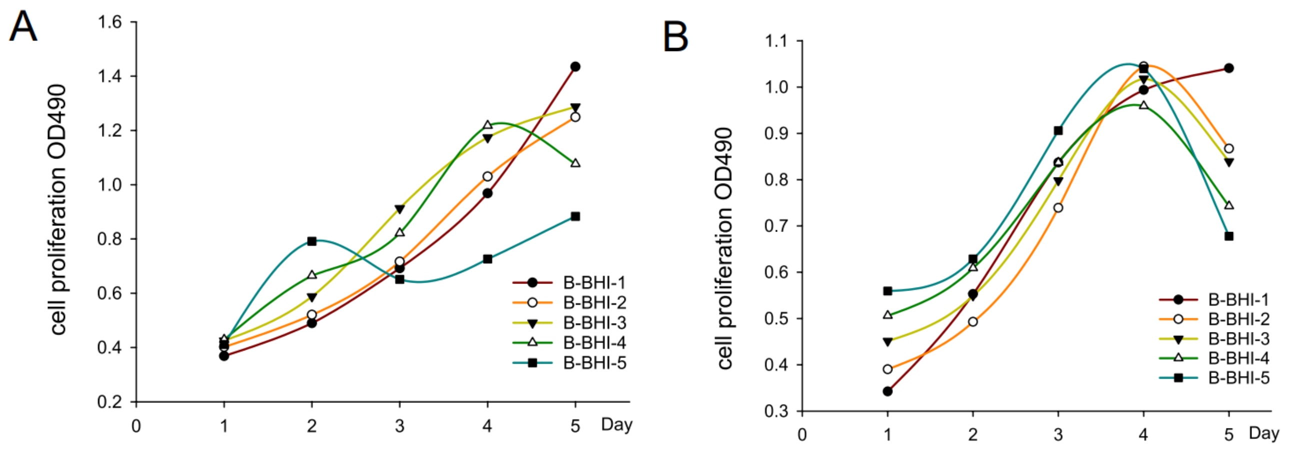

Fig. S8. MTT assay showed that varying concentrations of (A) *P. fungorum* and (B) *S. capitis* inhibited the proliferation of QBC939 cell line. B-BHI-(1-5) indicates the addition of different volumes (1-5 μl) of the supernatant of the bacterial solution, while the total volume of each group was ensured to be 5ul by the addition of PBS.

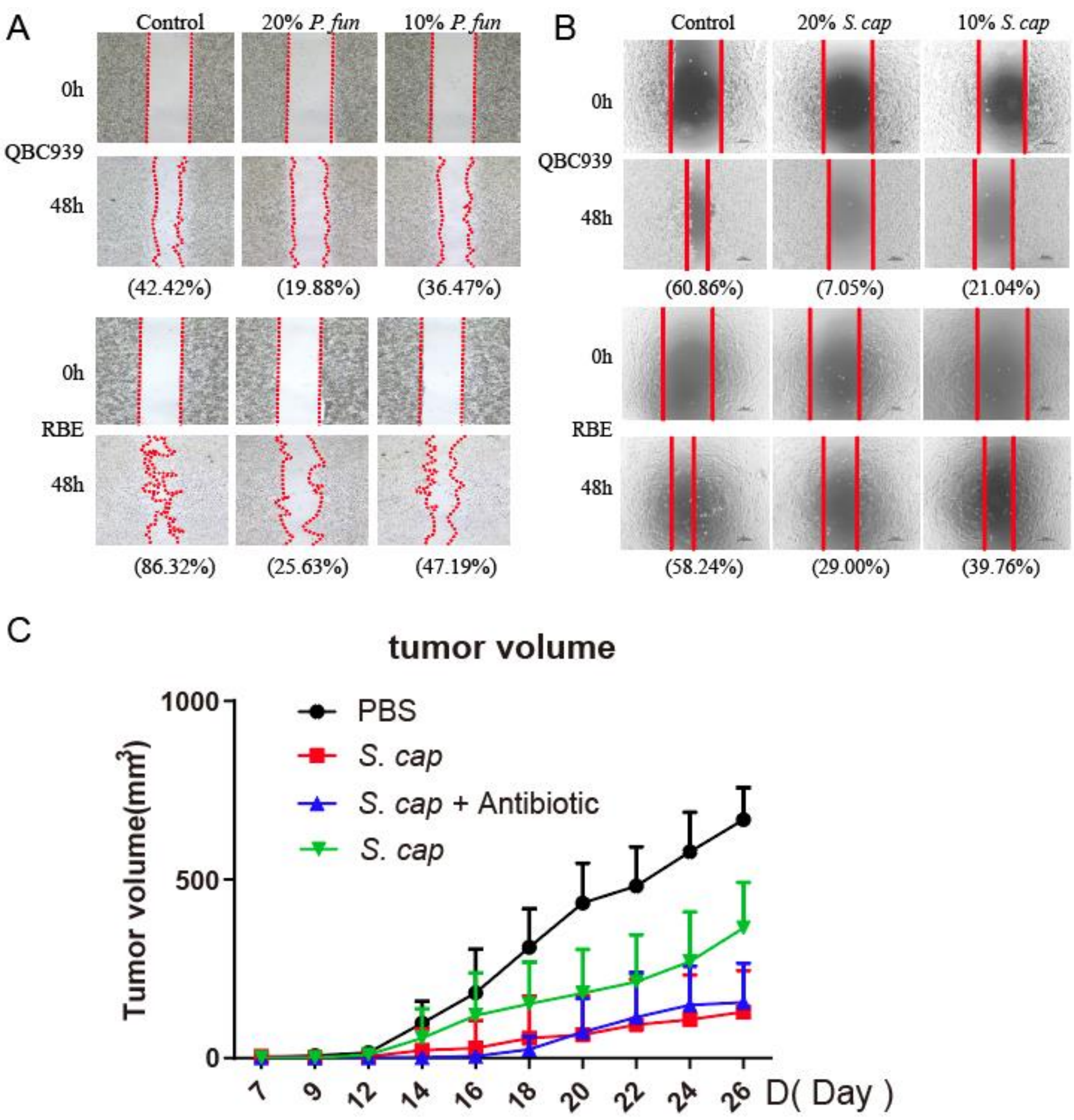

Fig. S9. Functional experiments related to *P. fungorum* and *S. capitis*. (A) The repeated scratch experiments of QBC939 (top panel) and RBE cells (bottom panel) with the addition of bacterial *P. fungorum* (*P. fun*) supernatant (no viable bacteria). The scratch assay (B) and mouse experiments (C) for *S. capitis* (*S. cap*), which served as a positive control in mouse experiments. Percentages in parentheses indicate the percentage of scratch healing.

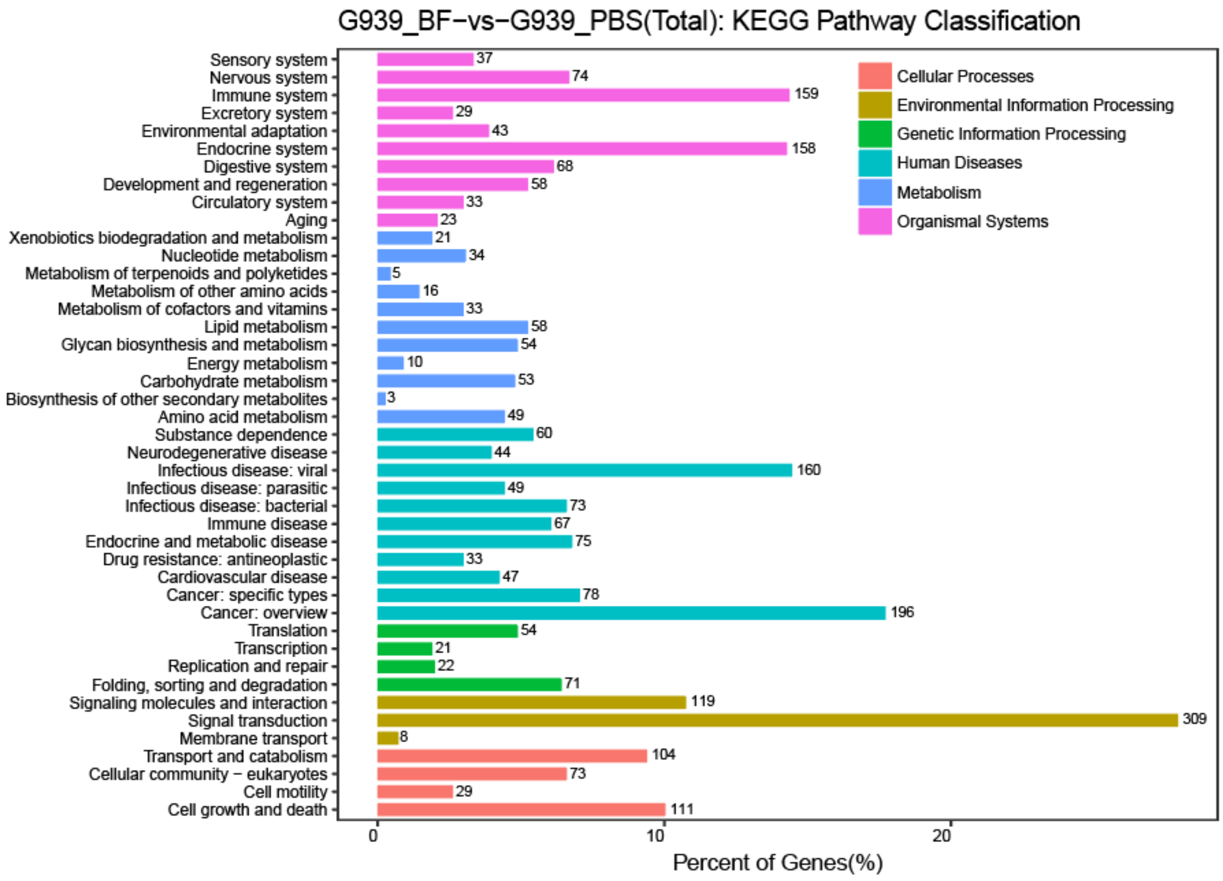

Fig. S10. KEGG pathway analysis of transcriptomic data from QBC939 cell line treated with bacterial supernatant.

References

1. Nejman D, Livyatan I, Fuks G, Gavert N, Zwang Y, Geller LT, et al. The human tumor microbiome is composed of tumor type-specific intracellular bacteria. Science 2020; 368:973-80.
2. Yang YW, Chen MK, Yang BY, Huang XJ, Zhang XR, He LQ, et al. Use of 16S rRNA Gene-Targeted Group-Specific Primers for Real-Time PCR Analysis of Predominant Bacteria in Mouse Feces. Applied and environmental microbiology 2015; 81:6749-56.
3. Sookoian S, Salatino A, Castano GO, Landa MS, Fijalkowky C, Garaycoechea M, et al. Intrahepatic bacterial metataxonomic signature in non-alcoholic fatty liver disease. Gut 2020; 69:1483-91.
